# UFObow: A single-wavelength excitable Brainbow for simultaneous multicolor ex-vivo and in-vivo imaging of mammalian cells

**DOI:** 10.1038/s42003-024-06062-3

**Published:** 2024-04-01

**Authors:** Jiahong Hu, Fangfang Yang, Chong Liu, Nengzhi Wang, Yinghan Xiao, Yujie Zhai, Xinru Wang, Ren Zhang, Lulu Gao, Mengli Xu, Jialu Wang, Zheng Liu, Songlin Huang, Wenfeng Liu, Yajing Hu, Feng Liu, Yuqi Guo, Liang Wang, Jing Yuan, Zhihong Zhang, Jun Chu

**Affiliations:** 1 Britton Chance Center and MoE Key Laboratory for Biomedical Photonics, Wuhan National Laboratory for Optoelectronics-Huazhong University of Science and Technology, Wuhan, Hubei 430074 China; 2 Guangdong Provincial Key Laboratory of Biomedical Optical Imaging Technology & CAS Key Laboratory of Health Informatics, Shenzhen Institute of Advanced Technology, Chinese Academy of Sciences, Shenzhen, 518055 China; 3 State Key Laboratory of Digital Medical Engineering, School of Biomedical Engineering, Hainan University, Haikou, Hainan 570228 China; 4 Biomedical Imaging Science and System Key Laboratory, Chinese Academy of Sciences, Shenzhen, 518055 China

**Keywords:** Fluorescence imaging, Genetic engineering

## Abstract

Brainbow is a genetic cell-labeling technique that allows random colorization of multiple cells and real-time visualization of cell fate within a tissue, providing valuable insights into understanding complex biological processes. However, fluorescent proteins (FPs) in Brainbow have distinct excitation spectra with peak difference greater than 35 nm, which requires sequential imaging under multiple excitations and thus leads to long acquisition times. In addition, they are not easily used together with other fluorophores due to severe spectral bleed-through. Here, we report the development of a single-wavelength excitable Brainbow, UFObow, incorporating three newly developed blue-excitable FPs. We have demonstrated that UFObow enables not only tracking the growth dynamics of tumor cells in vivo but also mapping spatial distribution of immune cells within a sub-cubic centimeter tissue, revealing cell heterogeneity. This provides a powerful means to explore complex biology in a simultaneous imaging manner at a single-cell resolution in organs or in vivo.

## Abstract (web summary)

UFObow is a single-wavelength excitable Brainbow technique, incorporating three newly developed blue-excitable fluorescent proteins. This method facilitates mapping of immune cells’ spatial distribution at a single-cell resolution.

## Introduction

Stochastic genetic labeling strategies with spectrally distinct fluorescent proteins (FPs) have been extremely useful to label multiple cells with different colors at once, greatly advancing our understanding of complicated biological processes^1^. Brainbow is one such strategy that uses Cre-lox recombination to achieve plenty of different hues by stochastic and combinatorial expression of three FPs^2^. To date, this technique has been widely exploited to map neuronal connections and track cell movement and fate in different model organisms including mouse^3–5^, zebrafish^6,7^, and fruit fly^8–10^. However, FPs in Brainbow possess different excitation spectra with peak difference greater than 35 nm, thus requiring multiple excitation lights, which cross-talk with emission channels for FPs. As a result, sequential fluorescence imaging with longer acquisition times has to be performed to avoid such cross-talk. Moreover, they are not spectrally well-compatible with other fluorescent dyes or FPs in the case of multicolor imaging.

Large-Stokes-shift FPs (LSS-FPs) (typically over 80 nm in emission peak difference) enable simultaneous multicolor imaging under single-wavelength excitation. To date, several UV-, blue- and cyan-excitable LSS-FPs have been developed^11–13^. However, UV light is phototoxic to mammalian cells, even under limited illumination, thereby limiting the use of UV-excitable LSS-FPs in living cells. Meanwhile, the cyan-excitable orange FP CyOFP1^14^, which is spectrally compatible with green FPs and cyan-excitable far-red FPs, has a redder excitation spectrum with peak at 497 nm. However, no cyan-excitable far-red FPs are available. On the contrary, blue-excitable FPs with different emission colors are available: cyan (ECFP variants^15,16^ and mTFP^17^), orange (LSSmOrange^18^), and far-red (LSSmKate^19^, mBeRFP^20^, and mKeima^21^). However, these FPs are not ideal for Brainbow due to the large spectral bleed-through between LSSmOrange and the other two classes of FPs (Supplementary Fig. 1), potentially reducing color complexity without bleed-through correction.

To address this issue, we engineered three new blue-excitable FPs with emission peaks at 488 nm, 566 nm, and 637 nm through directed evolution. Starting from these FPs, we also developed a single-wavelength excitable Brainbow, UFObow that features universal expression, fast imaging, and modular design. The UFObow has great color complexity without the bleed-through correction and high recombination efficiency in living cells. Moreover, we have demonstrated that UFObow can visualize the heterogeneity of metastatic tumor clones in vivo and the spatial distribution of immune cells in a sub-cubic centimeter tissue.

## Results

### Evolution of blue-excitable cyan, orange, and far-red FPs

To obtain blue-excitable cyan, orange and far-red FPs, we performed structure-guided mutagenesis of cyan mTFP^17^, orange mKOk^22^, and red Crimson^23^, respectively. These FPs are derived from different species with low sequence homology, thereby minimizing intermolecular recombination between FPs. Moreover, compared to ECFP variants, mTFP has reduced spectral bleed-through in the LSSmOrange channel due to its short emission tail. mKOk and Crimson are about 1.3 times brighter than mOrange (the progenitor of LSSmOrange) and mKate2 (the progenitor of LSSmKate and mBeRFP), making it possible to engineer bright blue-excitable orange and far-red FPs.

Recently, we developed a fast-maturating mTFP variant, mTFP2 (Supplementary Fig. 2), which is as bright as mTFP. However, mTFP2 is 2.3 times brighter than LSSmOrange, which could lead to decent photons from mTFP2 in the LSSmOrange channel. A close examination of the mTFP structure revealed that Thr63 is right below the phenolate ring of the chromophore (both protein termini facing upward) and tightly packs against the chromophore (Supplementary Fig. 3). We hypothesized that mutating Thr63 could make mTFP2 dimmer by increasing excited-state vibrations that could lead to non-radiative decay. Performing semi-saturated mutagenesis on Thr63 generated one dimmer and blue-shifted variant with the T63N mutation (Supplementary Fig. 4). The variant was named mTFP2b.

The excited-state proton transfer (ESPT) between the chromophore hydroxyl (proton donor) and its nearby residue (proton acceptor) is crucial for large Stokes shift in LSS-OFPs/RFPs^19^ (Supplementary Fig. 5). Especially, Asp or Glu at position 158 or 160 (numbering according to mKate2) in LSS-OFPs/RFPs contributes to ESPT. We reasoned that introducing Asp or Glu at position 158 or 160 in mKOk could create new LSS-OFPs (Supplementary Fig. 6). One round of library screening produced a dim blue-light excitable FP, LSSmKO0.1 (mKOk-V158D). To improve LSSmKO0.1’s brightness, we performed site-directed mutagenesis in a combinatorial and simultaneous manner at several locations at once, sampling alleles found naturally in OFPs and choosing sites that are likely to impact protein folding or chromophore brightness. After 16 rounds of screening, we exhaustively identified a bright variant containing 29 substitutions relative to its parent mKOk (Supplementary Fig. 4). We designated this final variant LSSmKOb.

Similar to LSSmKOb, a bright monomeric blue-excitable RFP, LSSmCrimson was developed from Crimson by introducing the C158D mutation and other beneficial mutations (Supplementary Fig. 6). However, there is quite some bleed-through between LSSmCrimson and LSSmKOb (Supplementary Fig. 7). It has been shown that the tandem green-red cross heterodimeric fluorescent protein GRvT, which exhibits high intramolecular Förster resonance energy transfer (FRET) efficiency, functions as an LSS-RFP^24^. Inspired by this study, we red-shifted the emission spectrum of LSSmCrimson by making a tandem fusion of LSSCrimson and the far-red FP dCardinal (cross dimers of LSSmCrimson and mCardinal^25^, respectively), both of which have dimerization mutations (T146M, D159N, R161A, H171I, K194F) (Supplementary Fig. 8). To improve its brightness, we optimized the loop connecting the chromophore of dCardinal by replacing the peptide NHTQG with KHPQD (Supplementary Fig. 8). The final variant was named as LSSfRFP.

**Fig. 1:**
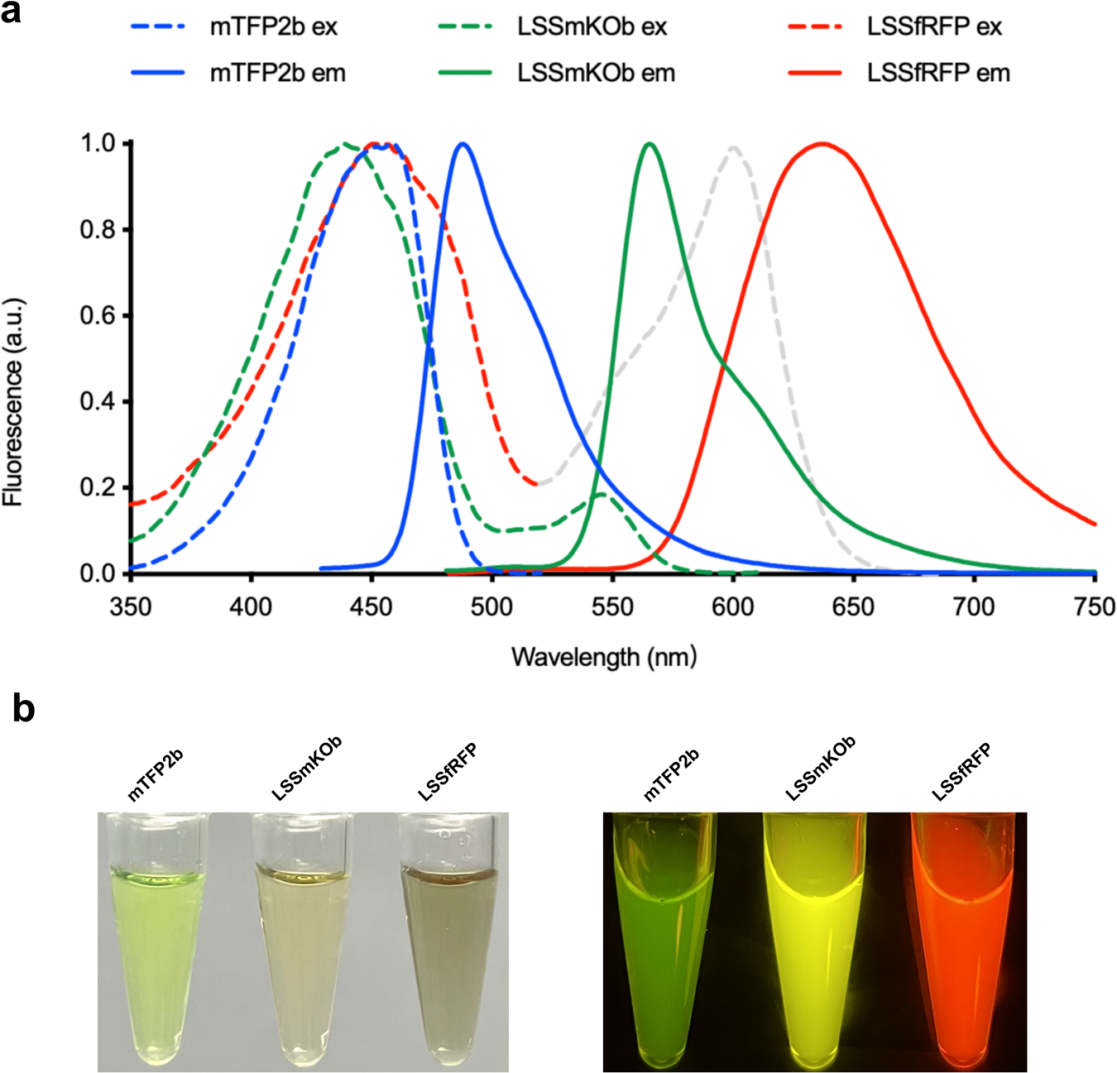
Spectral characteristics of mTFP2b, LSSmKOb and LSSfRFP. **a** Normalized excitation and emission spectra of FPs. The excitation spectrum of LSSmKOb has a shoulder at 545 nm. The excitation spectrum of dCardinal in LSSfRFP is shown as gray. **b** Transmittance (left) and fluorescence (right) of purified FPs. Fluorescence images were acquired with 400- to 500-nm excitation light and a yellow acrylic long-pass filter in a BlueView Transilluminator (Vernier).

Next, we examined fluorescence spectra and brightness of mTFP2b, LSSmKOb, and LSSfRFP in vitro. mTFP2b has a single excitation peak at 459 nm, whereas LSSmKOb has two excitation peaks: the major one at 437 nm (the protonated form with ESPT) and the minor one at 550 nm (the deprotonated form). LSSfRFP has two excitation peaks: 452 nm for LSSCrimson and 601 nm for dCardinal (Fig. 1, Supplementary Table 1). The emission maxima of mTFP2b, LSSmKOb, and LSSfRFP are 488 nm, 566 nm, and 637 nm, respectively (Fig. 1, Supplementary Table 1). Compared to mTFP, mTFP2b is blue-shifted by 4 nm in emission (Supplementary Fig. 6). LSSmKOb is bluer by 6 nm than LSSmOrange in emission. LSSfRFP is red-shifted by 23 nm in emission compared to LSSmCrimson (Supplementary Fig. 7). mTFP2b is 26% dimmer than mTFP, and LSSmKOb is 7.7% dimmer than LSSmOrange in molecular brightness. Compared to mTFP2b and LSSmKOb, LSSfRFP exhibits 2.6 and 4.8 times lower molecular brightness, respectively (Supplementary Table 1).

Finally, we examined FP’s maturation, aggregation behavior, spectral bleed-through, and photostability in living B16 cells under an inverted confocal fluorescence microscope equipped with a 405 nm laser (Supplementary Fig. 9). After 12 hours transfection, B16 cells expressing each FP exhibited bright fluorescence signal (Supplementary Fig. 10), indicating FP’s good maturation in living cells. Moreover, all FPs, unlike mCherry, formed very few puncta in living cells after long-term expression (5 days) (Supplementary Fig. 11). Consistent with the spectral characteristics in Supplementary Fig. 9, the spectral bleed-through occurred mainly in the LSSmKOb channel (553–602 nm): 17% of fluorescence in the mTFP2b channel (470–510 nm) for mTFP2b and 23% of fluorescence in the LSSfRFP channel (635–735 nm) for LSSfRFP (Supplementary Fig. 12). All FPs are relatively photostable: under 10-min laser illumination, mTFP2b, LSSmKOb, and LSSfRFP had a fluorescence decrease of 39%, 54%, and 62%, respectively, compared to 52% for mCerulean3, previously photostable cyan FP (Supplementary Fig. 13).

### Development and characterization of UFObow

**Fig. 2:**
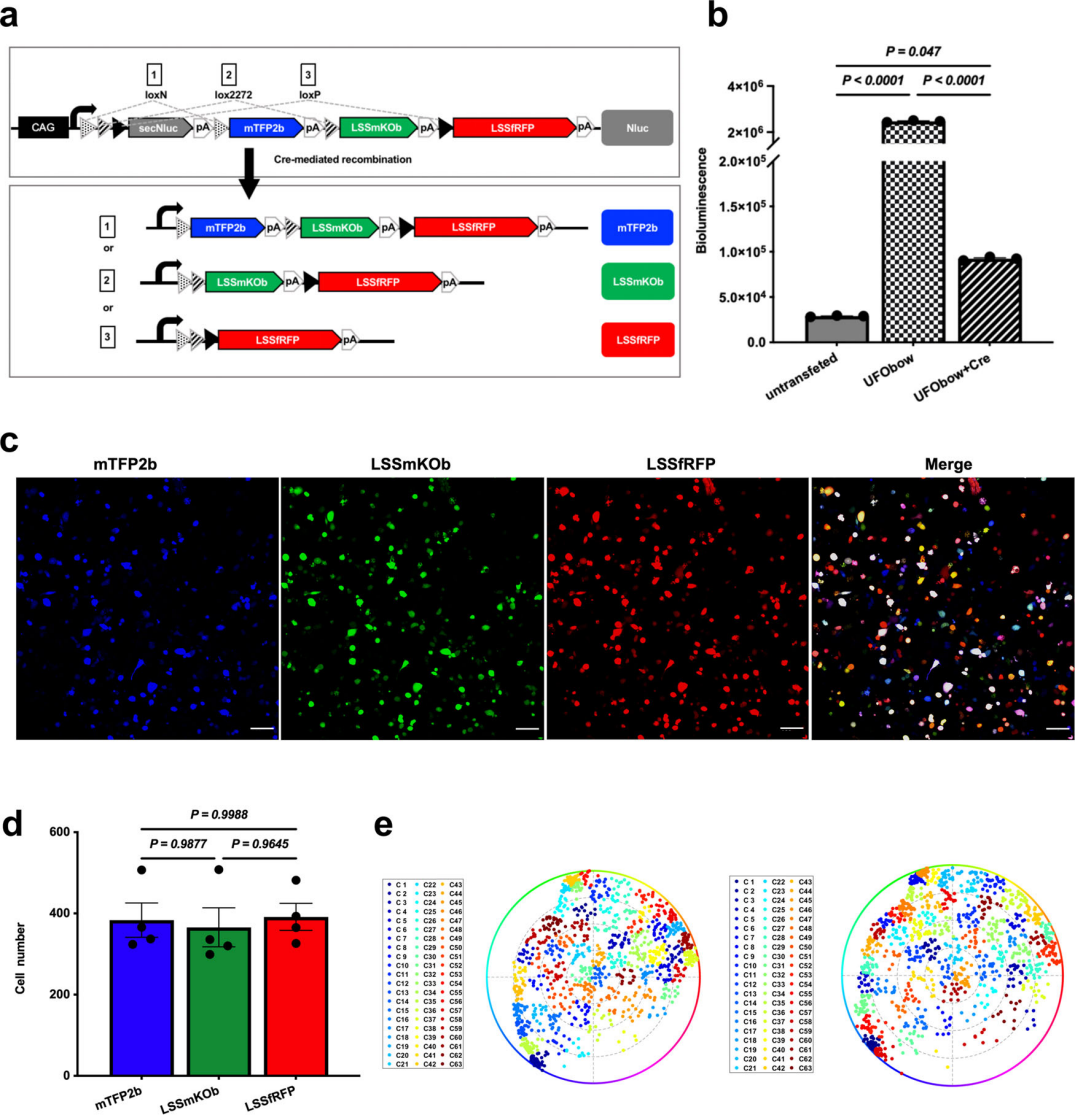
Characterization of UFObow in living HeLa cells. **a** Schematic diagram of the UFObow expression cassette including the CAG promoter. In the absence of Cre, only secNluc is expressed. In the presence of Cre, recombination occurs between one of the matched pairs of lox sites (loxN, lox2272 and loxP) and leads to irreversible selection of one of the FPs. **b** Bioluminescence detection of Nluc in HeLa cells expressing UFObow only or both UFObow and Cre. *P* values were calculated by one-way ANOVA with Tukey’s multiple comparisons post-test. **c** Fluorescence images of UFObow in HeLa cells. HeLa cells expressing UFObow and Cre were imaged using three emission collection channels: 470–510 nm for mTFP2b, 553–602 nm for LSSmKOb and 635–735 nm for LSSfRFP. Scale bar, 100 μm. **d** Numbers of HeLa cells expressing each FP: 384 ± 72 for mTFP2b, 366 ± 83 for LSSmKOb and 392 ± 57 for LSSfRFP. *P* values were calculated by one-way ANOVA with Tukey’s multiple comparisons post-test. Data are presented as mean ± SEM (*n* = 4 imaging fields). **e** Hue-Saturation (HS) plots of cell clusters. Each dot in the plot represents a cell, and dots with same color that is given by the clustering K-means algorithm are within a cluster. 63 clusters (C1-C63) were achieved from 1177 cells. The angle between a given dot and the center of the circle represents the hue (0-360°) and the distance from the center of the circle to a given dot represents the saturation (0-100%). In the presence of spectral cross-talk (left), the intra-cluster dispersion (SD) and inter-cluster distance (R) are 7.4% and 20.24%, respectively. In the absence of spectral cross-talk (right), the SD and R are 7.57% and 20.31%, respectively.

We started Brainbow3.2 as a starting point. In Brainbow3.2, three pairs of incompatible lox sites are concatenated, and recombine to create three mutually exclusive excision possibilities with the expression of a distinct FP each time upon Cre activation^26^. In addition, a non-fluorescent protein PhiYFP Y65A is inserted within the first lox pair to assess expression in the absence of Cre. However, this requires immunofluorescence of PhiYFP Y65A, which is laborious and time-consuming. On the contrary, bioluminescence detection of secreted Luciferase is an easy and fast way to report gene expression^27^. To make a single-wavelength excitable and easily detectable Brainbow, we first replaced original FPs mOrange2, EGFP, and mKate2 in Brainbow3.2 with new blue-excitable FPs mTFP2b, LSSmKOb, and LSSfRFP, and then changed PhiYFP Y65A to the highly bright secreted luciferase secNluc^28^. Moreover, to ensure broad expression in different tissues, we changed the tissue-specific Thy1 promoter to the universal CAG promoter. The resultant new Brainbow is named UFObow due to its universal expression (CAG promoter), fast acquisition (single-wavelength excitation), and modular design (replaceable expression cassettes) (Fig. 2a).

Next, we systematically examined recombination efficiency, recombination randomness, and color complexity of UFObow in mammalian cell lines co-expressing UFObow and Cre (Supplementary Fig. 14). Recombination efficiency is defined as the ability of Cre-mediated excision at all lox sites, which is determined by subtracting the percentage of secreted secNluc protein from 100%. This calculation assumes a linear relationship exists between the amount of secNluc DNA remaining uncut and the amount of secreted secNluc protein^29–31^. Recombination randomness is characterized as the probability of Cre-mediated excision at each pair of lox sites, which is measured as the number of cells expressing each FP. Color complexity is considered as the number of distinct cell clusters, each of which has its own color.

To determine the recombination efficiency of UFObow, we measured the bioluminescence (BL) signal of secNluc in the cell culture medium of tumor cell lines at 60 hours post-transfection because it is linearly proportional to the amount of secNluc protein in a certain concentration range (Supplementary Fig. 15). To determine the recombination efficiency of UFObow, we measured the bioluminescence (BL) signal of secNluc in cell culture medium of tumor cell lines at 60 hours post transfection. As expected, the experimental group (UFObow + Cre) had much lower BL signal than the control group (UFObow only). The calculated percentage of unexcised secNluc (a ratio of experimental to control groups) was 5.60% and 3.75% for HeLa cells and B16 cells, respectively (Fig. 2b and Supplementary Fig. 17b), indicating the recombination efficiency was greater than 94%. To rule out the possibility of false-positive BL signal caused by the cell itself, the culture medium from untransfected cells (the blank group) was checked out. Not surprisingly, the BL signal in the blank group was only 1.17% of that in the control group, suggesting cells themselves do not contribute to the BL signal.

To investigate the recombination randomness of UFObow, we quantified the numbers of tumor cells expressing each FP under transient transfection conditions. All three FPs were evenly distributed throughout both HeLa and B16 cells while the tetrameric far-red FP Katushka-S158A (a 112 kDa tetramer) was mainly localized in the cytoplasm, indicating the tandem-fusion FP LSSfRFP (53.9 kDa) is monomeric (Supplementary Fig. 18). The numbers for cells expressing each FP were comparable statistically (Fig. 2d and Supplementary Fig. 17c), indicating an equivalent recombination efficiency of Cre for each pair of lox sites.

To evaluate the color complexity of the UFObow, we generated color clusters in the HS (hue, saturation) color model by performing the K-means algorithm on FPs’ cellular brightness as described before^32^ (Supplementary Fig. 16). Given the fact that the spectral bleed-through occurs between LSSmKOb and the other two FPs, we asked whether such bleed-through affects the color complexity. 63 distinct color clusters were identified from 1177 HeLa cells with and without bleed-through correction (Fig. 2e), indicating that the bleed-through correction is not necessary when calculating the color clusters. However, the bleed-through correction causes some cells to change cluster assignment: 15% of ~2000 cells are re-classified into new clusters adjacent to the original ones. Therefore, the bleed-through correction is required in the lineage tracing studies. We also tested the UFObow’s color complexity in B16 cells and found 69 distinct colors from 1041 B16 cells (Supplementary Fig. 17d) in the absence of bleed-through correction. Taken together, these results indicate that UFObow can mark multiple cells with multiple, distinct colors in an efficient (high recombination efficiency), fast (single-wavelength illumination), and simple way (no bleed-through correction), making it useful when applied ex vivo and in vivo.

### UFObow enables efficient labeling of specific types of cells in vivo

Cells expressing specific molecules within a tissue are involved in specific functions or behaviors^33,34^. We asked whether UFObow can label specific types of cells in vivo. To this end, we first generated transgenic strains with multiple copies of the UFObow expression cassette by co-injection of plasmid DNA of UFObow and Tol2 transposase mRNA into zygotes (Supplementary Fig. 19), then performed successive rounds of incross to screen for UFObow mice with highest BL signal (Supplementary Fig. 20). No hepatocellular damage was detected according to the activities of hepatic marker enzymes aspartate aminotransferase (AST) and alanine aminotransferase (ALT) in serum (Supplementary Fig. 21). Three cell type-specific UFObow mice (transgenic mice expressing UFObow in specific cell types) were generated by crossing UFObow mice to three cell type-specific Cre mice (CX3CR1-Cre^35^, CD11c-Cre^36^, and Chat-Cre^37^). As a result, three cell type-specific UFObow mice were obtained: CX3CR1^cre^:UFObow, CD11c^cre^:UFObow, and Chat^cre^:UFObow, which drive UFObow expression in mononuclear phagocytes (MPs including monocytes, macrophages, and dendritic cells), CD11c^+^ dendritic cells (DCs), and cholinergic neurons, respectively.

**Fig. 3:**
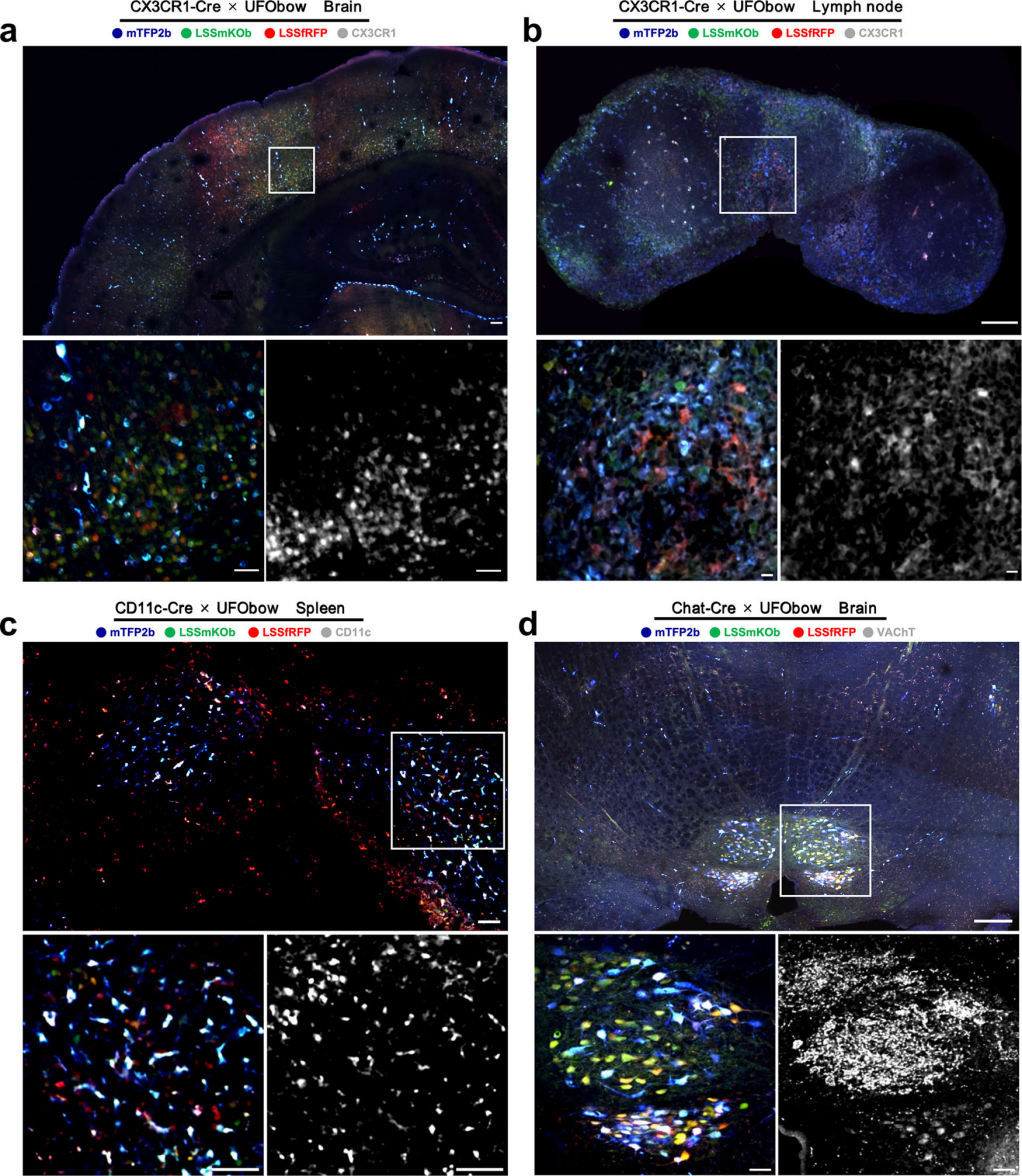
Multicolor imaging of specific types of cells in different tissues of transgenic mice co-expressing UFObow and Cre. Each panel consists of three fluorescence images: a tissue slice with FP fluorescence (top), and a region of interest (ROI, white box) of the slice with FP fluorescence (bottom left) and immunofluorescence (bottom right). **a**, **b** The representative fluorescence images of brain and lymph node slices of the CX3CR1^cre^:UFObow mouse. The PE anti-CX3CR1 antibody labeled CX3CR1-positive cells. Scale bar in the large image of **a**, 100 μm. Scale bar in the large image of **b**, 200 μm. Scale bar in small images, 50 μm. **c** The representative fluorescence images of a spleen slice of the CD11c^cre^: UFObow mouse. The Alexa Fluor 647 anti-CD11c antibody labeled CD11c-positive cells. Scale bar in the large image, 100 μm. Scale bar in small images, 50 μm. **d** The representative fluorescence images of a brain slice of the Chat^cre^:UFObow mouse. The Alexa Fluor 647 anti-VAChT antibody labeled VAChT-positive cells. Scale bar in the large image, 500 μm. Scale bar in small images, 50 μm.

Next, we examined the UFObow expression in organs that have abundant cell types mentioned above. Representative organs were collected as follows: brain and lymph nodes from the CX3CR1^cre^:UFObow mouse, spleen from the CD11c^cre^:UFObow mouse, and brain from the Chat^cre^:UFObow mouse. Confocal and two-photon imaging of organ slices (100 µm in thickness) showed that FPs were expressed with varied levels in all tested tissues, indicating the occurrence of Cre recombination (Fig. 3 and Supplementary Fig. 22). Then, we performed immunofluorescence of specific types of cells to verify the labeling specificity. As shown in Fig. 3a–d, confocal imaging demonstrated that UFObow-expressing cells colocalized with Phycoerythrin (PE) anti-CX3CR1 antibody-labeled microglia in the brain and MPs in the lymph node, Alexa Fluor 647 anti-CD11c antibody-labeled DCs in the spleen, and Alexa Fluor 647 anti-VAChT antibody-labeled cholinergic neurons in the brain (Supplementary Fig. 23).

Next, we examined how well UFObow color-codes cells in each tissue. To do that, a certain number of fluorescent cells from tissue slices were used to calculate the color complexity as described in the cultured cells. In the CX3CR1^cre^:UFObow mouse, 50-60 distinct colors were obtained in the brain (60 colors from 3006 cells) and lymph node (50 colors from 483 cells), respectively (Supplementary Fig. 24a, b). In the spleen of CD11c^cre^:UFObow mouse, 48 colors from 713 cells were generated (Supplementary Fig. 24c). In the Chat^cre^:UFObow mouse, 354 labeled cells from brain were categorized into 40 color groups (Supplementary Fig. 24d).

Overall, UFObow can be crossed with different cell-specific Cre mice to precisely label specific cell types in different organs with great color complexity and labeling specificity, providing a universal means to track multiple cells of a specific type ex vivo and in living mice.

### UFObow enables fast mapping of cell distribution in a sub-cubic centimeter tissue

Mapping the spatial distribution of a specific type of cells within a tissue is essential to understand the relationship between cell location and function^38–40^. To get a three-dimensional (3D) image of a sub-cubic centimeter tissue with single-cell resolution, thousands of images (or slices) with micrometer resolution in the axial (Z) direction and sub-micrometer resolution in the lateral (X, Y) direction are required. However, it would take several days to obtain all images with conventional Brainbow because sequential fluorescence imaging has to be performed (Supplementary Fig. 25).

UFObow could largely speed up the imaging process by taking advantage of being excited by a single wavelength. As a proof-of-concept, we attempted to map the spatial distribution of MPs in the left lateral lobe (16.5 × 11.3 × 5.3 mm^3^) of the liver from the CX3CR1^cre^:UFObow mouse using home-made cryo-fluorescence micro-optical sectioning tomography^41^. To obtain the entire dataset, the cyro-frozen liver was imaged (a twice Z stack imaging in 3 μm step) and then cut to remove the imaged part (a 6 μm-thick slice) by a knife alternatively. As a result, 1762 images (15.5 TB raw data) from 881 cryo-slices were collected with an imaging resolution of 0.32 μm × 0.32 μm × 3 μm in X × Y × Z.

**Fig. 4:**
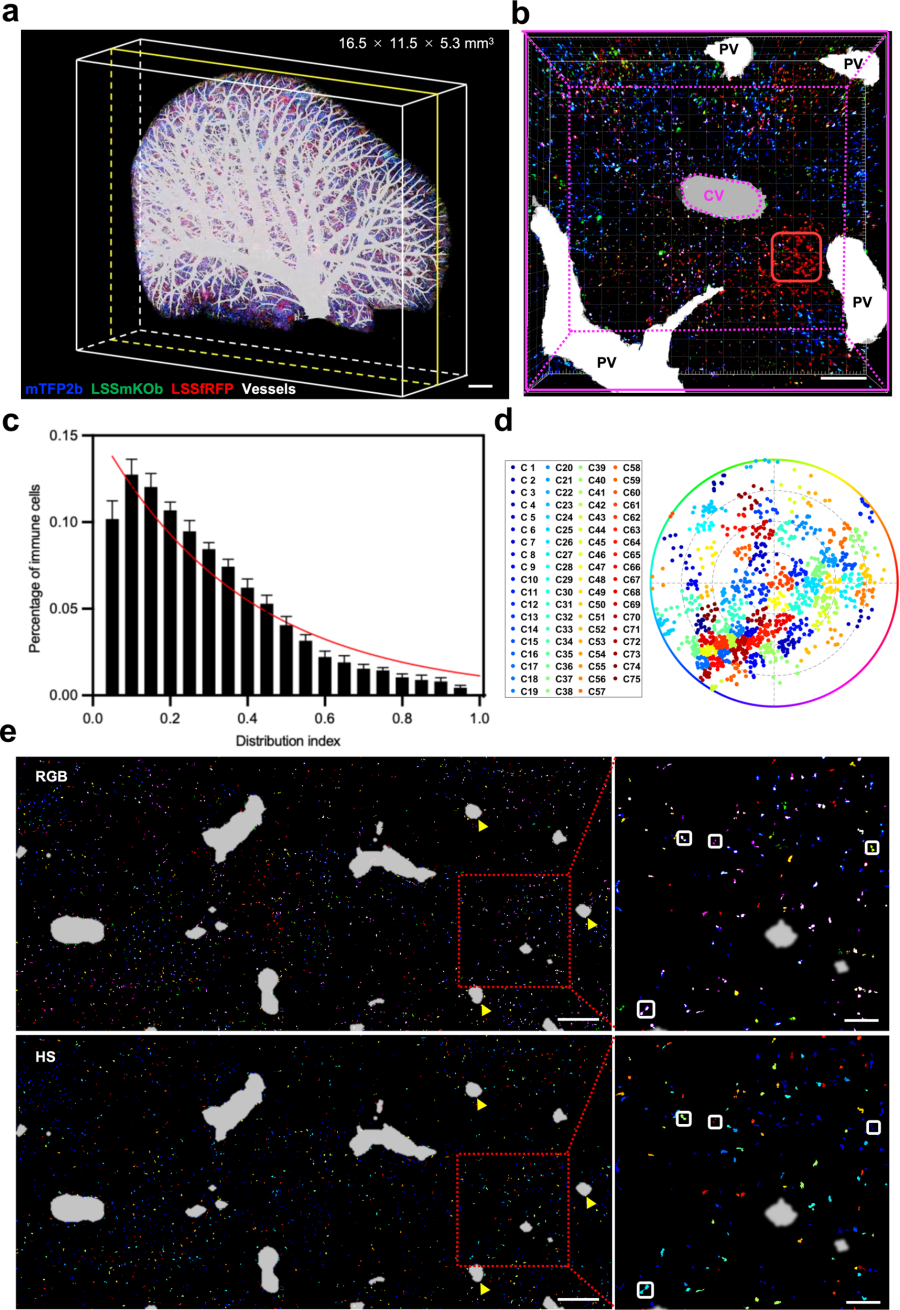
Spatial distribution of MPs in a mouse liver lobe. **a** A three-dimensional (3D) view of spatial distribution of MPs and blood vessels in the left lateral lobe of liver. The 3D reconstruction image is made of 1762 fluorescence images (881 slices) with an imaging resolution of 0.32 μm × 0.32 μm × 3 μm (X × Y × Z). Scale bar, 1000 μm. **b** A 3D view of a typical liver lobule. The boundary of the liver lobule is determined based on the location of PVs (white spots) and marked with yellow dashed line. The CV is marked with magenta dashed line. The red rectangle represents a cell group with similar color. Scale bar, 300 μm. **c** Quantitative analysis of spatial distribution of immune cells in liver lobules. The greater the distribution index is, the closer to CV an immune cell is. The width of each bar is 0.04 units of the distribution index. The trend line highlighted in red is fitted by the sigmoid function. Data are presented as mean ± SEM (n = 7 single images). **d** The HS plot of cell clusters for cells in (**a**). 75 clusters (C1-C75) were achieved from 1332 cells in 4 single images. The SD and R values are 7.67% and 20.08%, respectively. **e** Fluorescence images of combined six slices from (**a**). Top and bottom panels are RGB merge images (same as Figs. 2 and 3) and HS pseudo-color images (See Supplementary Fig. 19), respectively. The spectral cross-talk in (**e**) has been corrected. All CVs are marked with yellow arrows. Two adjacent MP cells with the same color are framed by a square box. Scale bar in the large images, 600 μm. Scale bar in the enlarged images, 100 μm.

Next, we reconstructed a 3D structure of the intact liver lobe including MPs and blood vessels. In each fluorescence image, fluorescent MPs were scattered as bright dots, whereas blood vessels appeared as dark spots under strong autofluorescence background (Supplementary Fig. 27a). In light of this fact and our previous study on 3D vascular architecture of intact mouse liver lobe^42,43^, we successfully segmented out hepatic veins (HVs) including its branch central veins (CVs), portal veins (PVs), and MPs, and created a liver lobe’s 3D structure showing the spatial locations of MPs and blood vessels (Fig. 4a, Supplementary Movie 1). To better characterize the spatial location of MPs, a typical hepatic lobule, the building block of the liver tissue, was built up based on the locations of CV and PV (Fig. 4b). Quantitative analysis of 7 images from 7 different hepatic lobules showed that MPs enriched around the Portal triad (PT) rather than the CV (32.0% versus 1.2%), and the density of MPs was negatively correlated with the distance from the CV in the hepatic lobule (Fig. 4c), suggesting the cell number heterogeneity of MPs in the liver.

Lastly, we explored the clustering characteristics of MPs in the liver. The color complexity analysis on 1332 cells from randomly selected 4 slices showed that 75 cell color-clusters existed in the liver lobe (Fig. 4d), suggesting that the origins of MPs in the liver were differentiated into different lineages. Again, the spectral cross-talk correction did not change the color complexity (Supplementary Fig. 28). Interestingly, regions close to the PT exhibited several cell groups with similar color in each group, suggesting the location heterogeneity of clustered MPs in the liver. Moreover, color-coded images of a single slice or multiple slices clearly showed some adjacent MPs appeared to be in the same color-cluster (Fig. 4e and Supplementary Fig. 27b), suggesting cell proliferation from the same clonal lineage rather than two independent cells during or after differentiation. This is because multiple copies of UFObow expand the color complexity and thus significantly lower the probability that two independent cells would randomly have the same HS score and cluster assignment^44^. Therefore, UFObow-based cell-distribution mapping provides a powerful tool for visualizing not only the spatial arrangement of cells but also their differentiation and proliferation within a large-volume liver.

### Multi-color imaging of the heterogeneity of tumor metastases with UFObow

Heterogeneity is a distinctive feature of tumor growth and metastasis and is largely affected by the tumor environment (TME) such as immune cells and blood vessels^45–47^. Therefore, tracking the tumor heterogeneity in vivo is important to understand how tumor cells colonize and grow, and how they interact with the TME. To do so, we tracked tumor metastases in an experimental liver metastasis model. We first generated a stable B16 cell line co-expressing UFObow and Cre (UFObow-B16) using the piggyBac transposase. The UFObow-B16 cells showed rich hues with a color complexity of 45 under 382 tested cells (Supplementary Fig. 29a, b). In particular, the encoded colors of UFObow-B16 cells appeared to be consistent after cell division and replication (Supplementary Fig. 29c), suggesting that all cells in a clone originating from a single tumor cell are identical in color. Then, we monitored the growth dynamics of tumor clones in a liver metastasis mouse model^48,49^ created by intrasplenic injection of UFObow-B16 cells.

**Fig. 5:**
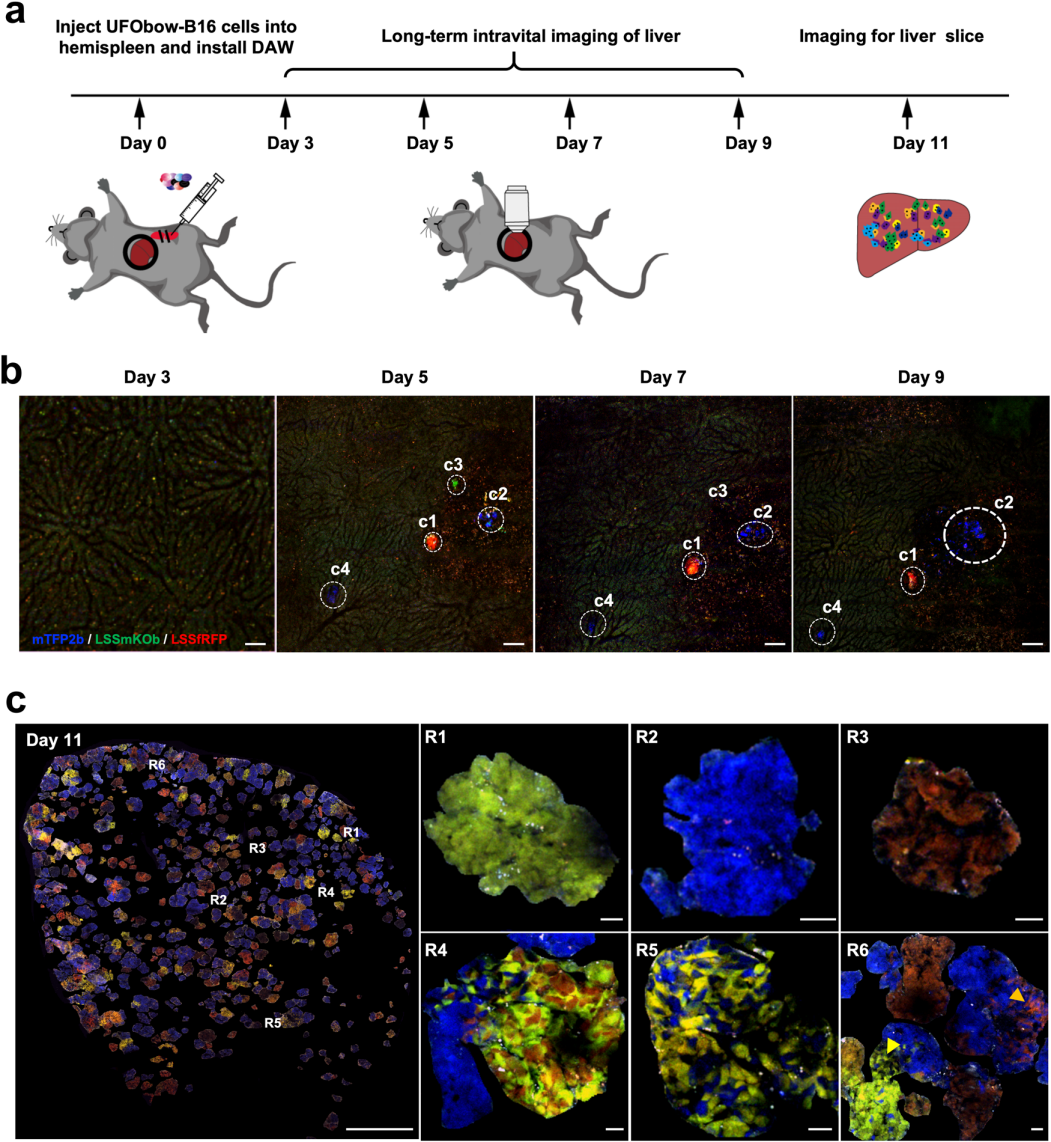
Multicolor imaging of the clonal growth of liver tumor metastases labeled with UFObow. **a** Schematic diagram of long-term intravital imaging (Day 3 – Day 9) of liver metastasis through a drawer-type abdominal window model and fluorescence imaging of liver slices (Day 11). The DAW is indicated by a black circle. **b** Time-series of fluorescence images of UFObow-B16 metastases in the mouse liver. Four clones (c1-c4) of UFObow-B16 metastases with different colors were monitored from Day 3 to Day 9. Scale bar, 50 µm. **c** Fluorescence images of liver tumor metastases on Day 11. The regions R1-R6 were enlarged for showing single-color (R1-R3) and multi-color (R4-R6) coded metastases. The orange and yellow arrows indicate intra- and inter-tumor communications between subclones. Scale bar in the large image, 500 µm. Scale bar in enlarged images (R1-R6), 10 µm. The spectral cross-talk in **b** and **c** has been corrected.

The process of tumor cell colonization and colony formation in vivo was monitored through a liver window^48,50^ for 9 days under an inverted confocal microscope equipped with a 405-nm laser (days 3, 5, 7, and 9) (Fig. 5a). In the first 3 days, tumor cells in the liver did not form metastases because tumor cells were stuck in hepatic sinusoids and entered into the space of Disse (Fig. 5b). Four tumor metastatic foci with different colors were observed on day 5 (Fig. 5b) and exhibited different growth dynamics on the following four days: clone #1 (c1) became bigger and then smaller, clone #2 (c2) kept growing, clone #3 (c3) disappeared, clone #4 (c4) became smaller and then bigger. These results suggested that at early (tumor cell colonization) and middle (colony formation) stages during liver metastasis, tumor cells grow as monoclonal populations and metastatic foci exhibited different cell fates: growing or fading away.

To verify whether the heterogeneity of metastasis clones increased at a late stage, we performed a large-field fluorescence imaging to visualize metastatic foci in mouse liver 11 days after UFObow-B16 cell injection. Metastatic foci were dense and presented two growth patterns: single color and mixed color (Fig. 5c). As shown in Fig. 5c, single-color tumor clones dispersed their respective cells and exchanged with other clones, which resulted in intratumoral invasion and clonal intermixing. Thus, our results indicate that the tumor colonization and outgrowth are predominantly monoclonal at the early and middle stages, and different metastasis foci were considerably intermixed at the late stage.

To visualize tumor clones and their microenvironment (TME), the immune cells were labeled with spectrally UFObow-compatible fluorescent dyes or FPs in the UFObow-B16 liver metastasis model. We injected near-infrared (NIR) nanopomegranate^51^ into mice with liver metastasis of UFObow-B16 cells to in vivo label blood-derived monocyte-macrophages through the tail vein. Interestingly, different tumor clones recruited different numbers of immune cells (Supplementary Fig. 30). We also made liver slices from mT/mG transgenic mouse^52^ (blood vessels and hepatic cells are labeled with tdTomato) with liver metastasis of UFObow-B16 cells and stained liver slices with Alexa Fluor 647 anti-CD3 antibody to mark CD3^+^ T cells. Confocal imaging of liver slices showed that dense vascularity at the tumor margins as well as CD3^+^ T cells infiltrating the tumor interior (Supplementary Fig. 31).

Taken together, UFObow, along with advanced optical microscopy and multi-color labeling method, is useful for investigating the heterogeneity of tumor clones and immune cells ex vivo and in vivo, which facilitates understanding of complicated biology.

## Discussion

In this study, we have created a single-wavelength excitable Brainbow, UFObow, which consists of three new blue-excitable FPs with decent photostability and appropriate brightness. UFObow exhibits high recombination efficiency and great color complexity in living cells. Using UFObow, we have successfully tracked the tumor heterogeneity in living mice, confirming intrinsic communications among tumor subclones. Furthermore, we have demonstrated the heterogeneity of immune cells in cell number and spatial location in the liver by multicolor 3D imaging of the liver lobe with UFObow.

Engineering single-wavelength excitable FPs with distinct emission colors but little spectral bleed-through has been challenging due to FPs’ intrinsic chromophore structures and wide emission spectra. Through extensive structure-guided mutagenesis, we developed two new LSS-FPs, LSSmKOb and LSSfRFP, which exhibit a larger emission peak difference than LSSmOrange and the reddest LSS-RFP mKeima (71 nm versus 48 nm). Moreover, mTFP2b is blue-shifted compared to mTFP, reducing the spectral bleed-through between mTFP and LSSmKOb. Thus, the color complexity of UFObow is maintained even in the absence of spectral bleed-through correction. To our best knowledge, this is the first study to report the development of three FPs for three-color imaging with a single-wavelength excitation.

By taking advantage of cell-specific UFObow mice and cyro-fMOST imaging, we demonstrated the localization heterogeneity of clustered MPs in the liver. There are two possible explanations for such heterogeneity. One explanation is the different origins of differentiated MPs. Most MPs arise from erythro-myeloid progenitors (EMPs) in the yolk sac and hematopoietic stem cells (HSCs) in the bone marrow. EMP-derived MPs differentiate before birth and thus likely exhibit very few cell clusters. In contrast, HSC-derived MPs generate quite a few cell clusters as they mature and differentiate throughout a lifetime^53^. However, this heterogeneity can also be explained by the local proliferation of EMP-derived MPs, some of which retain proliferative after embryonic development and replenish the hepatic macrophages near the PT even under steady-state conditions^54,55^.

The differentiation and proliferation of tissue-resident cells remain poorly understood in various organs, such as kupffer cells in the liver, resident cardiac macrophages (rcMacs) in the heart, microglia in the brain, and red pulp and white pulp macrophages in the spleen^56–58^. We anticipate that UFObow imaging would reveal more differentiation and proliferation patterns of tissue-resident cells. However, some Cre strains express Cre recombinase in non-target cells and tissues^59^, which can confound experimental interpretation. To avoid this problem, conditional Cre mouse strains like CreER can be used to improve labeling specificity in a given time window^60^.

Multicolor imaging of liver metastases expressing UFObow confirmed that tumor cells tend to grow as monoclonal populations at the early stage and then intermix at the late stage during the tumor progression, which is consistent with the previous study^61–63^. Furthermore, we first successfully traced the growth and spontaneous regression of tumor clones for several days in vivo. Using this model, it would be interesting to see how different tumor clones respond variably to the local pathophysiological microenvironment or a given drug^64^. However, it is still unclear what causes such differences at the molecule level in each clone. In the future, UFObow imaging along with spatial transcriptomics would address this question.

In summary, the new Brainbow derivative reported here, UFObow, should be useful whenever fast imaging is desired. Besides cell tracking, UFObow should also be useful for cell lineage tracing and neuron mapping, and can be also extended to other model organisms including Zebrafish, which have been realized in Brainbows.

## Methods

### Mutagenesis and screening of libraries

Site-directed mutagenesis was performed by overlap-extension PCR using PrimeSTAR Max DNA Polymerase (R045A, Takara, China). All PCR products were ligated into a constitutive bacterial expression vector pNCS containing T7 promoter using the In-Fusion HD enzyme (638909, Takara, China). The Stellar competent cells (Clontech, USA) were used for cloning and protein expression. Libraries of mutants were incubated overnight at 34 °C on LB agar plates and maintained thereafter at room temperature for 4~6 hours. Fluorescence was imaged on plates. Colonies expressing mutants were screened for fluorescence in a dark box with a white Mi-LED fiber-optic light source (Edmund, USA), a 405/40-nm excitation filter, three emission filters (530/30 nm for mTFP2b, 580/20 nm for LSSmKOb, 630/40 nm for LSSfRFP), and an MDK 41 BU02 CCD camera controlled with Micro-Manager 1.4.21 (NIH). Bacterial colonies of interest were patched on LB agar plates and incubated overnight at 34°C. For LSSmKOb and LSSfRFP, the brightest FP in each round was chosen for the subsequent round of mutagenesis.

### Characterization of FPs in vitro

Fluorescent proteins with polyhistidine tags were expressed from pNCS vectors in Stellar bacterial cells, purified with cobalt-chelating affinity chromatography (Pierce) and desalted into phosphate-buffered saline (PBS) pH 7.2 using gel filtration columns (Bio-Rad, USA). Excitation spectra and emission spectra were measured with an Infinite M1000 fluorometer (Tecan, Switzerland). Extinction coefficients (Ecs) were calculated using the base-denaturation method^14^. For LSSfRFP, the EC value presented in Supplementary Table 1 is 2 times of calculated EC value because both dCardinal and LSSdCrimson in LSSfRFP equally contribute to absorption at 446 nm (denatured form). Quantum yields (Qys) were determined using mTFP (QY = 0.85), LSSmOrange (QY = 0.45), and LSSmCherry1 (QY = 0.29) as standards.

### Plasmid construction

pC3-puro-pCAG-ASAP1-emcvIRES-mCherry is a gift from Dr. Michael Lin at Stanford University. PB-H2B-mCherry and Pbase are gifts from Dr. Xiaohui Wu at Fudan University, China.

pcDNA3.1-mTFP2b, pcDNA3.1-LSSmKOb, pcDNA3.1-LSSfRFP were constructed by subcloning mTFP2b, LSSmKOb and LSSfRFP from pUFObow into pcDNA3.1(+) vector using BamHI and EcoRI enzymes.

Mammalian expression plasmid pUFObow was generated based on pThy1-Brainbow3.2 (#45179, Addgene, USA). Briefly, the promoter Thy1 was replaced with the universal promoter CAG, the non-fluorescent protein PhiYFP Y65A was replaced with the secreted version of luciferase Nluc (secNluc), and the fluorescent proteins mOrange2, EGFP, and mKate2 were replaced with the mTFP2b, LSSmKOb and LSSfRFP. Because FPs’ ends have similar DNA sequences, pUFObow was made by a series of cloning steps. First, pC3-puro-pCAG-ASAP1-emcvIRES-mCherry was cut by EcoRI, EcoRV and AflIII enzymes to obtain a linear vector (pCAG) including a CAG promoter and an ampicillin resistance gene. Second, the secNluc, LSSmKOb, mTFP2b and LSSfRFP fragments were amplified from pNL1.3.CMV-secNluc (JQ437372, Promega, China), pNCS-LSSmKOb, pNCS-mTFP2b, and pNCS-LSSfRFP, respectively. Third, Lox sites, WPRE, and polyA were amplified from pThy1-Brainbow3.2, and then assembled with secNluc, LSSmKOb, mTFP2b and LSSfRFP by overlap-extension PCR to generate four large fragments. Finally, these four large fragments were sequentially subcloned into pCAG using In-Fusion HD enzyme.

To construct PB-UFObow, we cloned the whole UFObow expression cassette except the CAG promoter to PB-H2B-mCherry. First, we added two cleavage sites AvrII and I-CeuI into PB-H2B-mCherry by amplifying mCherry (AflII--AvrII--mCherry--I-CeuI--EcoRI) and cloning it back to PB-H2B-mCherry using AflII and EcoRI sites to generate PB-H2B-mCherry-new. Second, pUFObow was digested with I-CeuI and AvrII to generate the UFObow cassette without the CAG promoter, followed by subcloning the UFObow fragment into PB-H2B-mCherry-new using I-CeuI and AvrII sites.

All primers in the above cloning are shown in Supplementary Table 2.

### Tumor cell lines

HeLa and B16F10 (B16) melanoma cell lines were obtained from ATCC and the BOSTER company (Wuhan, China), respectively. The UFObow-B16 cell line was obtained with the Piggybac transposon system. Briefly, B16 cells were co-transfected with PB-UFObow, pCMV-Cre, and Pbase plasmids. After two weeks culture, transfected cells were resuspended in PBS (HyClone, USA) and then sorted for fluorescent cells by a cell sorter (MoFlo XDP, Beckman) with a 488-nm laser and three emission collection channels (FITC, RPE-TR, and RPE-Cy5 for mTFP2b, LSSmKOb, and LSSfRFP, respectively). Sorted Cells were cultured for another week to obtain the stable cell line UFObow-B16.

Cell lines were maintained in RPMI-1640 (HyClone, Utah, USA) (B16 and UFObow-B16) or DMEM/high glucose (HyClone, USA) (HeLa) containing 10% FBS (Gibco, New York, USA) and penicillin/streptomycin (both at 100 units/mL) in a humidified incubator (Thermo, Massachusetts, USA) at 37 °C with 5% CO_2_.

### Mice

C57BL/6 mice were obtained from Hunan Slack King of Laboratory Animal Co., Ltd (Changsha, China). B6J.B6N(Cg)-Cx3cr1^tm1.1(cre)Jung^/J (CX3CR1-Cre, JAX: 025524), B6.Cg-Tg(Itgax-cre)1-1Reiz/J (CD11c-Cre, JAX: 008068), B6;129S6-Chat^tm2(cre)Lowl^/J (Chat-Cre, JAX: 006410) and B6.129(Cg)-Gt(ROSA)26or^tm4(ACTB-tdTomato,-EGFP)Luo^/J (mT/mG, JAX: 007676) mice were purchased from the Jackson Lab (Bar Harbor, ME, USA). UFObow knock-in mice were generated by injecting the transgenic vector pTol2-UFObow containing the UFObow expression cassette and Tol2 arms), and Tol2 transposase mRNA into zygotes (Biocytogen, Beijing, China), and then screened strains with high bioluminescence signal by several successive rounds of incross. UFObow mice are available to the academic research community through Jackson Laboratory (JAX Stock 038448). To generate CX3CR1^cre^:UFObow, CD11c^cre^:UFObow and Chat^cre^:UFObow mice, CX3CR1-Cre, CD11c-Cre and Chat-Cre mice were crossed with UFObow mice, respectively. All mice were bred at the animal facility of WNLO-HUST under specific-pathogen-free (SPF) conditions and used at 6-10 weeks old. All animal experiments were approved by the Animal Experimentation Ethics Committee of Huazhong University of Science and Technology (IACUC Number: 843). We have complied with all relevant ethical regulations for animal use.

### Bioluminescence detection of UFObow

For cultured cell lines, B16 and HeLa cells were transfected with pUFObow or cotransfected with pUFObow and pCMV-Cre using Lipofectamine 2000 (Invitrogen, 11668019). After 60 hours transfection, 95 μL of culture medium was transferred from the culture bottle to a 96-well white plate (5085649, Molecular Device, USA). 5 μL of 200 μM h-Coelenterazine solution (40906ES08, Yeason, China) was added into the sample well to detect bioluminescence signal with multifunctional enzyme label instrument (FlexStation 3, Molecular Devices, USA).

For transgenic mice, 50 μL of blood was collected into a tube containing 10 μL of 12 mg/mL EDTA solution after cutting off 1-2 cm tail and then centrifuged for 10 min at 3000 rpm to get the serum. Next, 95 μL of reaction buffer (PBS/8.8 mM EDTA/1% (v/v) NP-10) and 5 μL of serum were sequentially added into a well of 96-well white plate. 5 μL of 200 μM h-Coelenterazine solution was added into the sample wells to detect the bioluminescence signal as mentioned above.

### Determination of cross-talk factor

First, pcDNA3.1-mTFP2b, pcDNA3.1-LSSmKOb, and pcDNA3.1-LSSfRFP were transfected into B16 cell line separately. After 48 hours transfection, confocal imaging was performed using a 405 nm laser and three emission collection channels for each FP (470–510 nm, 553–602 nm, and 635–735 nm that are defaults for mTFP2b, LSSmKOb, and LSSfRFP, respectively). All fluorescence signal was subtracted from the background. The cross-talk factor of a specific channel for a specific FP is calculated as the ratio of the fluorescence signal in the specific channel to the fluorescence signal in its default channel.

### Photobleaching of FPs in living cells

pcDNA3.1-mTFP2b, pcDNA3.1-LSSmKOb, and pcDNA3.1-LSSfRFP were transfected into B16 cells separately. mCerulean3-B16 cell line was established in our lab^65^. After 60 hours transfection, live cell photobleaching measurements were performed on fluorescent B16 cells using an inverted confocal fluorescence microscope (FV3000, Olympus, Japan) equipped with a dry 20× NA0.8 objective, a 405 nm laser, four emission collection channels (470–510 nm for mCerulean3, 470–510 nm for mTFP2b, 553–602 nm for LSSmKOb, and 635–735 nm for LSSfRFP). Time-series images were acquired every 16 s under 10-minute continuous illumination.

### Live cell confocal imaging

B16 and HeLa cells were cotransfected with pUFObow and pCMV-Cre. After 60 hours transfection, cells were plated onto a chambered coverslip with 8 wells (ibidi, Germany) and cultured for 12 hours before imaging. Live cell confocal imaging was performed with an inverted confocal fluorescence microscope (FV3000, Olympus, Japan) equipped with a dry 20× NA0.8 objective, a 405 nm laser, and four emission collection channels (470–510 nm for mCerulean3, 470–510 nm for mTFP2b, 553–602 nm for LSSmKOb, and 635–735 nm for LSSfRFP).

### Immunofluorescence

Fresh mouse tissues were soaked in 4% paraformaldehyde (PFA) for 10 hours and then sectioned at a thickness of 100 μm by a vibrating blade microtome (Leica VT1200, Germany). Then, slices were blocked in PBS containing 0.02% Triton X-100 (Sigma, Missouri, USA) and 1% bovine serum albumin (BSA) for 1 hour and then incubated with a directly-labeled antibody overnight at 4 °C. Primary antibodies used in this study were as follows: PE anti-CX3CR1 (Clone SA011F11 Cat# 149005, BioLegend), Alexa Fluor 647 anti-CD11c (Clone N418 Cat# 117312, BioLegend), and Alexa Fluor 647 anti-CD3 (Clone 17A2 Cat# 100209, BioLegend). For indirectly-labeled antibody staining, the slices were blocked as described above, then incubated with goat anti-mouse VAChT (Clone ABN100, Sigma) for 1 hour at room temperature, and further stained with the secondary antibody donkey anti-goat IgG(H + L) (Clone A21447, Biolegend) overnight at 4 °C. After washing off the unbound antibody, samples were enclosed using PBS containing 50% glycerol (w/w). Samples were imaged under an inverted confocal fluorescence microscope (FV3000, Olympus, Japan) with dry 10× NA0.4 and dry 20× NA0.8 objectives.

### Liver metastasis model

The liver metastasis model was established as described^66^. Briefly, C57BL/6 mice were anesthetized with a mixture of 10 mg/kg xylazine and 100 mg/kg ketamine hydrochloride (Sigma, USA). Under anesthesia, an incision was made in the upper left-hand side of the abdomen and then the spleen was taken out carefully. One-half of the spleen ligated was inoculated with 5×10^5^ tumor cells and was resected after 5 minutes inoculation. Lastly, the incision was sutured. After surgery, mice were kept on a heated plate (Thermo Plate, TOKAI HIT, Shizuoka-ken, Japan) to maintain body temperature at 37 °C till complete recovery.

### Intravital imaging of livers

Intravital liver imaging was performed either through a drawer-type abdominal window (DAW) (Fig. 5b)^50^ or on an exposed liver (Fig. 5c). For liver imaging with DAW, mice were assembled with DAW during preparing the liver metastasis model. Nanopomegranate was prepared as described before^51^ and injected into mice *via* tail vein 2 hours before imaging. Mice were anesthetized with a mixture of 10 mg/kg xylazine and 100 mg/kg ketamine hydrochloride and then immobilized in custom-made boxes, exposing the imaging window^48^. For liver-exposed imaging in vivo, the abdominal cavity of anesthetized mouse was depilated, and then the liver was exposed and pasted on a cover glass.

The intravital imaging was performed using an inverted confocal fluorescence microscope (FV3000, Olympus, Japan) with a dry 20× NA0.8 objective. Images were taken every 17 s for at least 1 hour. The excitation lasers and emission collection channels are as follows: 640 nm/720–800 nm for DiR, 405 nm/470–510 nm for mTFP2b, 405 nm/553–602 nm for LSSmKOb, 405 nm/635–735 nm for LSSfRFP, and 561 nm/570–620 nm for tdTomato. Intravital imaging of liver metastases was performed on days 3/5/7/9 after tumor cell inoculation.

### Generation of RGB merge images and HS pseudo-color images for cells expressing UFObow

For RGB merge images, raw fluorescence images were first subtracted from the background, uncorrected or corrected for the spectral cross-talk, and then imported into Fiji or MATLAB for generating RGB images. For Fiji, click “Image-->Color-->Merge channels…”. Images of mTFP2b, LSSmKOb, and LSSfRFP are assigned to the blue channel, green channel, and red channel, respectively when performing “Merge channels”. For MATLAB, create a multi-channel RGB image. Images of mTFP2b, LSSmKOb, and LSSfRFP are assigned to the blue channel, green channel, and red channel. The spectral cross-talk correction is described in the following equations:

$$\,{{GV}}_{{mTFP}2b}={{GM}}_{{BFP}}$$

$${{GV}}_{{LssfRFP}}={{GM}}_{{RFP}}$$

$${{GV}}_{{LssmKOb}}=100 \% \times {{GM}}_{{KFP}}-17 \% \times {{GM}}_{{BFP}}-23 \% \times {{GM}}_{{RFP}}$$

Where GV_mTFP2b_, GV_LSSmKOb_ and GV_LSSfRFP_ represent the spectral cross-talk corrected fluorescence intensity of mTFP2b, LSSmKOb and LSSfRFP, respectively. GM_BFP_, GM_KFP_ and GM_RFP_ represent uncorrected fluorescence intensity of mTFP2b, LSSmKOb and LSSfRFP, respectively. 17% and 23% are the spectral cross-talk coefficients for mTFP2b and LSSfRFP in the LSSmKOb channel, respectively.

For HS pseudo-color images, the data processing in detail is described in Supplementary Fig. 26.

### Multicolor imaging of a whole liver lobe, 3D reconstruction, and spatial distribution analysis

The CX3CR1^cre^:UFObow mouse anesthetized was first perfused with 0.1 M PBS (pH 7.0) followed by 4% paraformaldehyde (PFA). Then, the liver was soaked in 4% PFA for fixation followed by 30% sucrose solution for dehydration at 4°C. The left lateral lobe of the liver was isolated and then embedded in O.C.T (Sakura Finetek USA.Inc, USA) and frozen with liquid nitrogen before imaging. The liver lobe was imaged using home-made cryo-fluorescence micro-optical sectioning tomography^41^. The imaging module was equipped with a line scanning microscope, an objective lens (LUCPLFLN 20×, NA = 0.45, Olympus, Japan), a 450 nm CW diode laser (LWBL450-100mW, Laserwave Optoelectronics Technology, China), and three emission filters (FF01-709/167-25 for LSSfRFP, FF01-575/59-25 for LSSmKOb, and FF01-488/50-25 for mTFP2b, Semrock, USA). Line scanning was achieved by moving the tissue at a uniform speed of 250 mm/min along the X-axis. Z stack imaging was achieved by moving the Z-axis stage. After a twice Z stack imaging in a 3 μm step, the frozen sample was moved toward a knife to cut the imaged part and expose the underlying surface with a thickness of 6 μm (2 images/slice). The imaging and cutting were repeated alternately until the entire dataset (1762 images with 15.5 TB raw data in total) was acquired. The imaging area of the microscope is constantly adjusted according to the cross-sectional area of the liver to save imaging time. The imaging time for each slice varies from 10 seconds (the smallest area of liver with 4.8 × 2.3 mm^2^) to 190 seconds (the largest area of liver with 16.5 × 11.3 mm^2^). The overall imaging time excluding the slice cutting and manual operation is 31.3 hours.

The 3D reconstruction is completed with four steps. First, 3D continuous images without overlap of three channels are stitched and registered from raw fluorescence images according to the recorded imaging area of each slice in Python (3.9.7, Python Software Foundation). Second, Otsu algorithm is used to segment fluorescent cells from all slices in MATLAB (https://www.mathworks.com). Third, the blood vessels are contrast-enhanced, segmented, and reconstructed using the region growth algorithm in Amira software (http://www.fei.com/software/amira-avizo). Fourth, three-dimensional reconstruction and rendering of the cell and blood vessel data are performed in Imaris software (https://imaris.oxinst.com). The data-processing time for step 1, step 2,  step 3 and step 4 is 21 hours, 3 hours, 12 hours, and 2 hours, respectively.

The distribution index (µ) is defined as the ratio of D2 to D, where D2 is the distance from a given immune cell to its nearest boundary of a liver lobule, D is the distance from the CV to the nearest (relative to a given immune cell) boundary of a liver lobule.

### Color complexity

The image analysis is mainly completed in Fiji (https://fiji.sc/Fiji) and MATLAB (https://www.mathworks.com) softwares via multiple steps. Data processing in detail is described in Fig. S1. The rationality of the number of color clusters is verified based on the intra-cluster dispersion (SD) and inter-cluster distance (R) that are calculated using Euclidean distance. The Euclidean distance of an N-dimensional (N-D) space is defined as follows:

$${{{{{\rm{d}}}}}}\left(x,y\right)=\sqrt{\mathop{\sum }\limits_{i=1}^{N}{\left({x}_{i}-{y}_{j}\right)}^{2}}$$

Where d (x, y) is the Euclidean distance, x and y are two points in an N-D space, and i is its i-th dimension.

The intra-cluster dispersion of a given cluster C_i_ can be defined as the sum of the distances from the points within the cluster to the centroid.

$${{{{{\rm{i}}}}}}{ntra}({C}_{i})=\mathop{\sum}\limits_{x\in {C}_{i}}{{{{{\rm{d}}}}}}\left(x,{c}_{i}\right)$$

Where c_i_ is the centroid of cluster C_i_.

Similarly, the inter-cluster distance between two clusters can be measured by the average of the distances between all sample points in the two clusters.

$${inter}({C}_{i},{C}_{j})=\mathop{\sum}\limits_{i\in {C}_{i},j\in {C}_{j}}d(i,j)/(\left|{C}_{i}\right|* \left|{C}_{i}\right|)$$

Where d (i, j) is the distance between point i and point j.|C_i_| and |C_j_| represent the number of points in cluster C_i_ and cluster C_j_.

It is noted that the greater the ratio of R to SD (R/SD) is, the better the clustering is. In general, the R/SD value must be greater than 1^67^. The criteria for effective color clustering is SD < 8% and R > 20%.

### Crystal structures of FPs

All FP structure figures were generated using PyMol (version 2.5.4, Schrödinger, LLC, Portland, USA) without raying.

### Statistics and reproducibility

Experimental data are presented as means ± SEM. For the comparison of two groups, an unpaired *Student*’s t-test was used. For the comparison of three or more groups, a one-way ANOVA test followed by Tukey’s multiple comparisons test was used. All statistical analyses were performed using GraphPad Prism 9 (trial version). Differences between or among groups are denoted as ns for not significant, * for *P* < 0.05, ** for *P* < 0.01, *** for *P* < 0.001, and **** for *P* < 0.0001.

### Reporting summary

Further information on research design is available in the Nature Portfolio Reporting Summary linked to this article.

### Supplementary information

Supplementary Information

Description of Additional Supplementary Files

Supplementary Data 1

Supplementary Movie 1

Reporting Summary

## Data Availability

The DNA sequences of mTFP2b, LSSmKOb, LSSfRFP and UFObow have been deposited in GenBank with the following accessions: PP277508, PP277507, PP277506, and PP277509, respectively. The pUFObow plasmid has been deposited in the China Center for Type Culture Collection under the accession M2023066. Source data related to plots and graphs in this study can be found in Supplementary Data 1. Other data are available upon reasonable request to the corresponding authors.

## Code availability

The codes for computing the color complexity are available at https://github.com/chongliu01/UFObow.
