# Supplementary material for: UFObow: A single-wavelength excitable Brainbow for simultaneous multicolor ex-vivo and in-vivo imaging of mammalian cells
Source: Commun Biol. 2024 Apr 1;7:394. doi: 10.1038/s42003-024-06062-3 (PMC10984974; doi:10.1038/s42003-024-06062-3)
Supplement: Supplementary file 1 — Supplementary Information [file 42003_2024_6062_MOESM1_ESM.pdf]

UFObow: a single-wavelength excitable Brainbow for simultaneous multicolor *ex-vivo* and *in-vivo* imaging of mammalian cells

Jiahong Hu[1,†], Fangfang Yang[2,†], Chong Liu[1,†], Nengzhi Wang[1,†], Yinghan Xiao[2,†], Yujie Zhai[1], Xinru Wang[1], Ren Zhang[1], Lulu Gao[1], Mengli Xu[3], Jialu Wang[1], Zheng Liu[3], Songlin Huang[3], Wenfeng Liu[2], Yajing Hu[1], Feng Liu[2], Yuqi Guo[2], Liang Wang[2], Jing Yuan[1,*], Zhihong Zhang[1,3,*], Jun Chu[2,4,*]

[1] Britton Chance Center and MoE Key Laboratory for Biomedical Photonics, Wuhan National Laboratory for Optoelectronics-Huazhong University of Science and Technology, Wuhan, Hubei 430074, China

[2] Guangdong Provincial Key Laboratory of Biomedical Optical Imaging Technology & CAS Key Laboratory of Health Informatics, Shenzhen Institute of Advanced Technology, Chinese Academy of Sciences, Shenzhen 518055, China

[3] State Key Laboratory of Digital Medical Engineering, School of Biomedical Engineering, Hainan University, Haikou, Hainan 570228, China

[4] Biomedical Imaging Science and System Key Laboratory, Chinese Academy of Sciences, Shenzhen 518055, China

† These authors contributed equally.

*Correspondence should be addressed to: yuanj@hust.edu.cn or czyzzh@mail.hust.edu.cn or jun.chu@siat.ac.cn

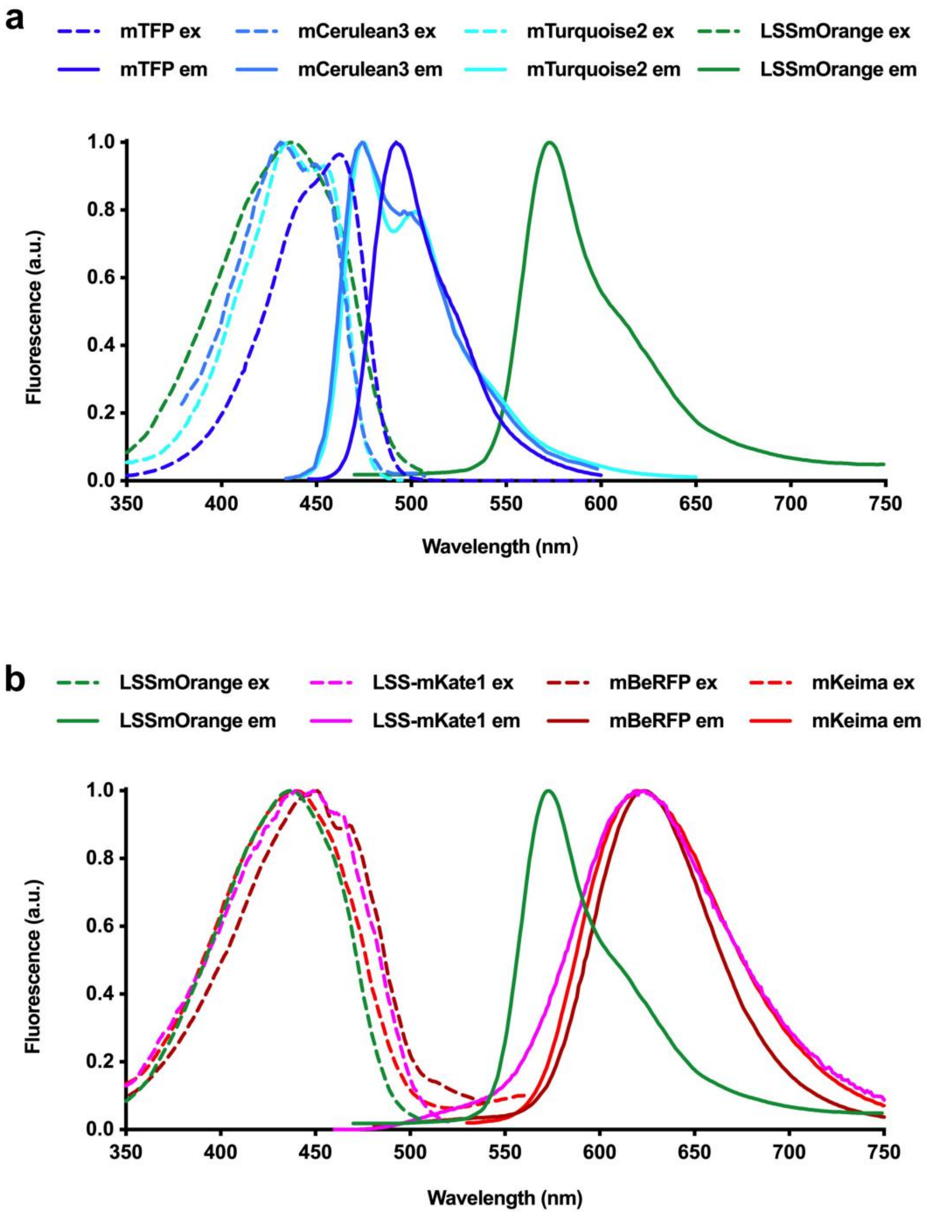

Supplementary Figure 1 Fluorescence spectra of cyan-excitable cyan, orange and far-red FPs.

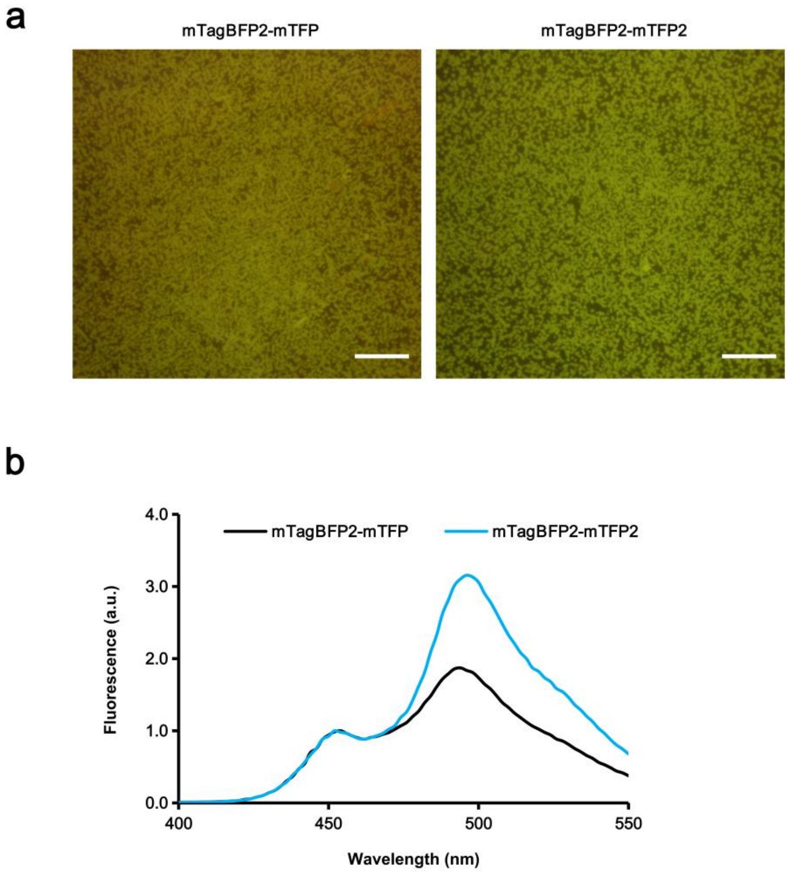

Supplementary Figure 2 Maturation of mTFP2 and mTFP. (a) Fluorescence images of bacteria expressing tandem fusions: mTagBFP2-mTFP and mTagBFP2-mTFP2. Fluorescence imaging was taken 24 hours after transformation. Scale bar, 1 cm. **(b)** Emission spectra of tandem fusions in (a).

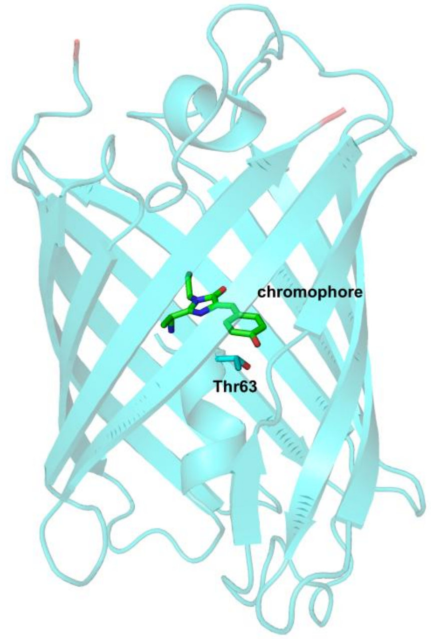

Supplementary Figure 3 Thr63 and the chromophore of mTFP. The crystal structure of mTFP (PDB: 2otb) is shown as cartoon. Thr63 and the chromophore are shown as stick. Protein termi are highlighted in red.

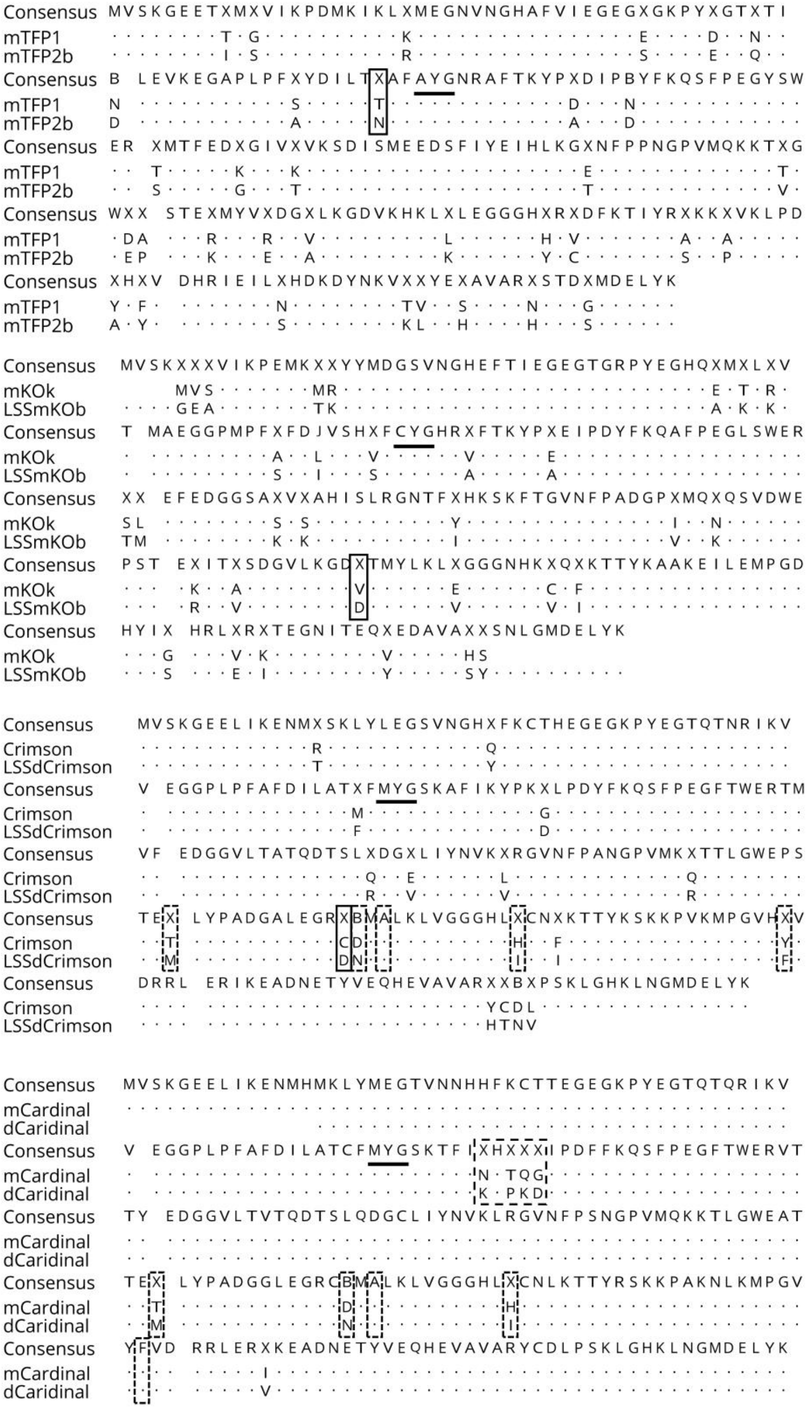

Supplementary Figure 4 Protein alignment of FP variants. Chromophore-forming residues are underlined. All mutations mentioned in this study are shown in rectangles (solid for large stokes shift, square dot for dimerization, dash for loop optimization).

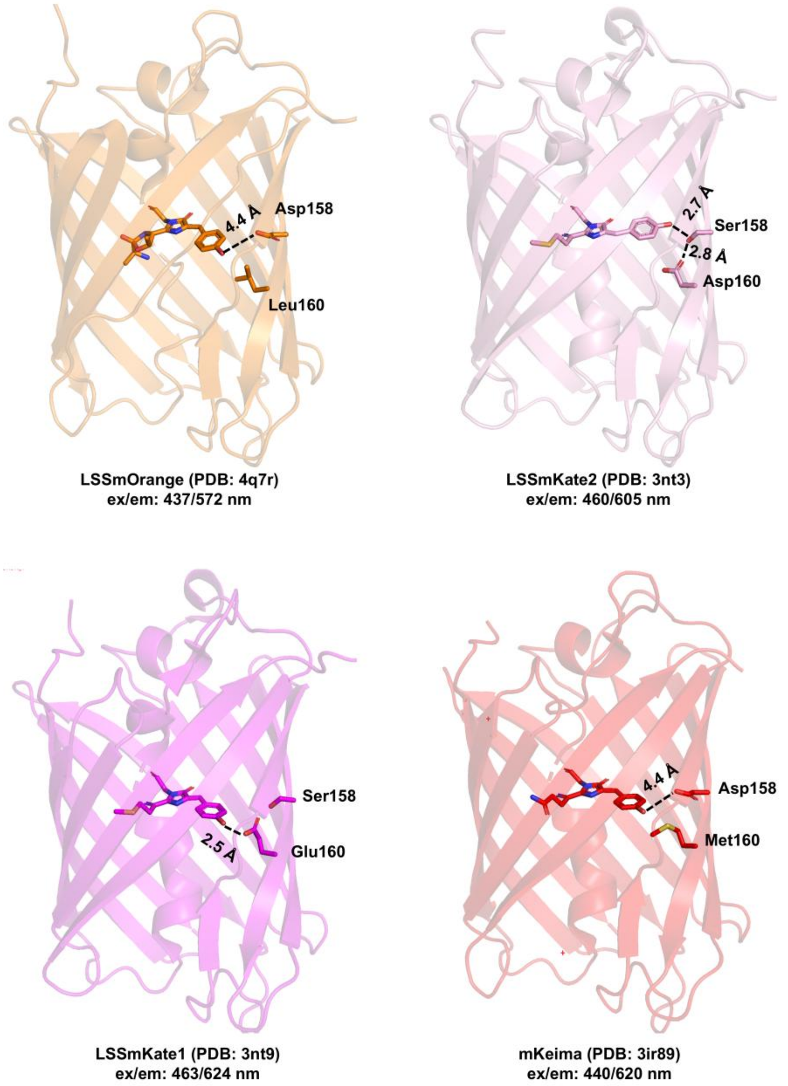

Supplementary Figure 5 ESPT in LSS-OFPs and LSS-RFPs. All crystal structures are shown as cartoon. Chromophores and residues at positions 158 and 160 are shown as stick.

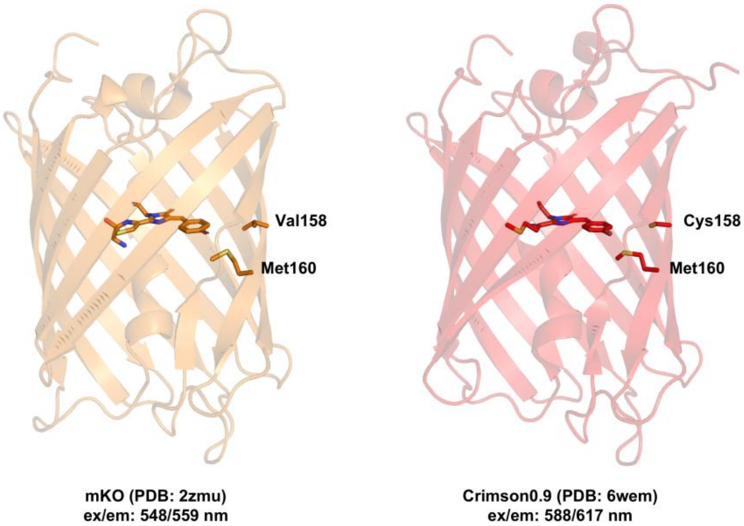

Supplementary Figure 6 Positions 158 and 160 in mKO and Crimson0.9. All crystal structures are shown as cartoon. Chromophores and residues at positions 158 and 160 are shown as stick. mKOk and Crimson are derived from mKO and Crimson0.9, respectively. They have similar fluorescence spectra and almost same residues at key positions including 158 and 160.

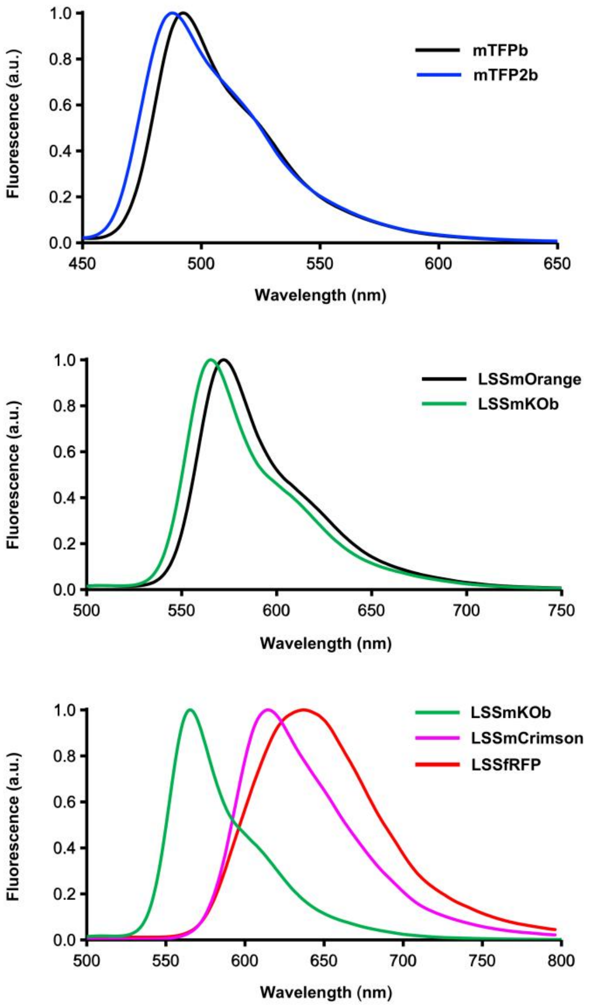

Supplementary Figure 7 Emission spectra of different FPs.

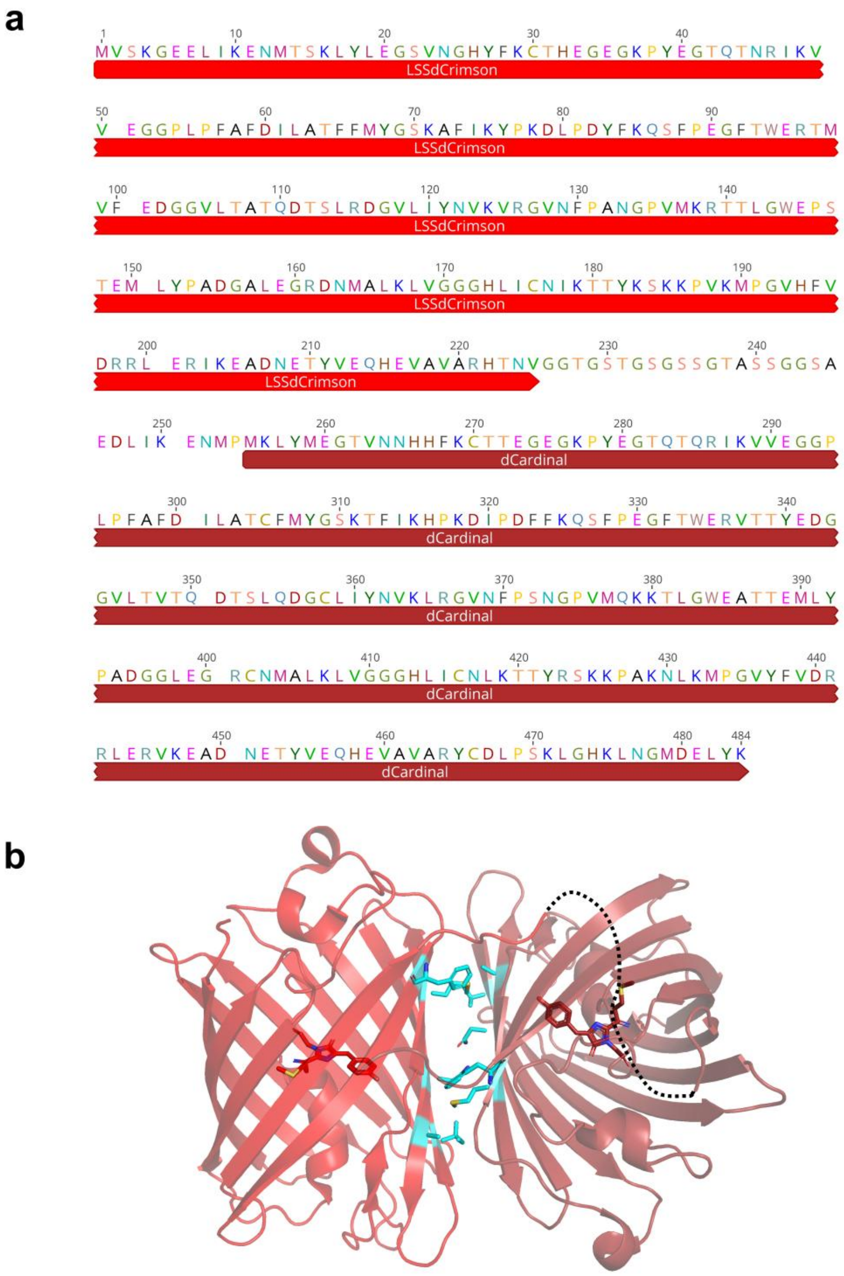

Supplementary Figure 8 Protein sequence and modelled structure of LSSfRFP. (a) Chromophore-forming residues are underlined. All mutations mentioned in this study are shown in rectangles (solid for large stokes shift, square dot for dimerization, and dash for loop optimization). **(b)** Predicted structure of LSSfRFP. Mutations in the A-C dimerization interface are shown in cyan. The link between LSSdCrimson (left) and dCardinal (right) is shown as dotted line.

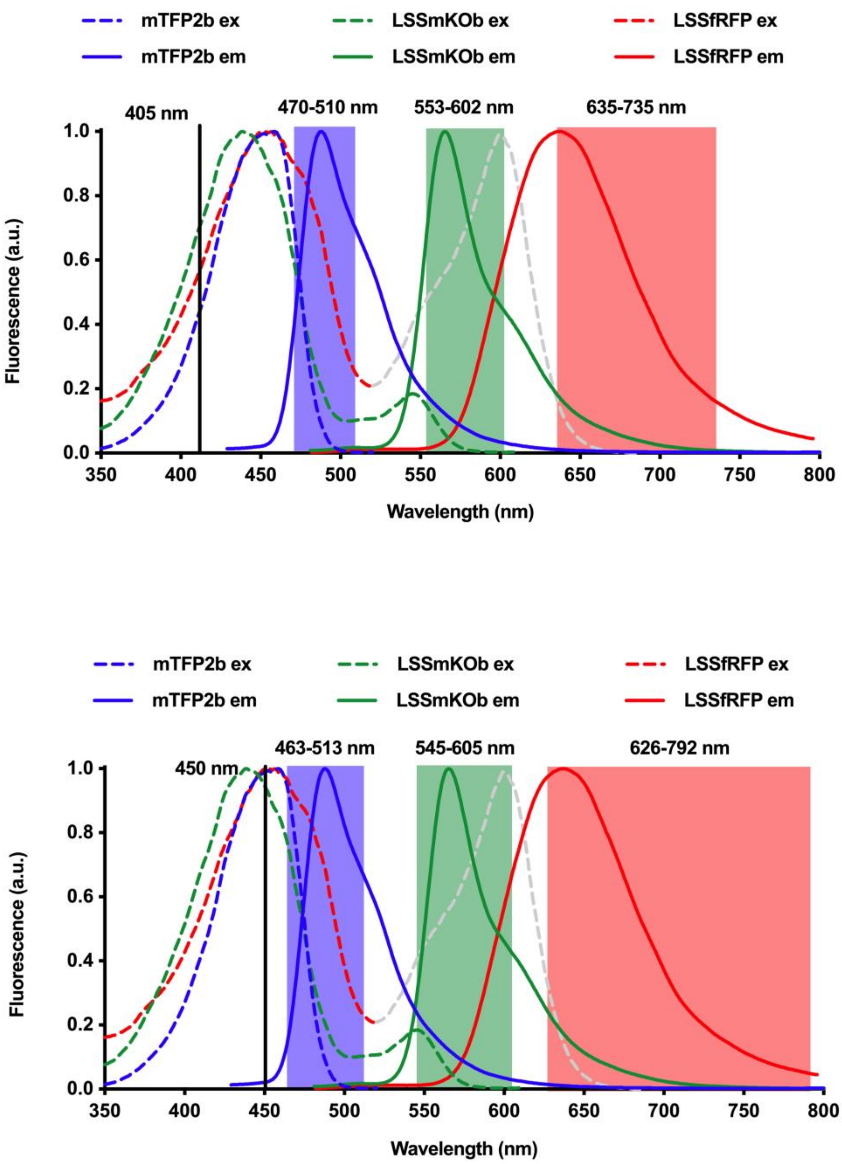

Supplementary Figure 9 Excitation lines and emission collection channels for confocal imaging of UFObow in this study. Top and bottom panels are for all experiments except 3D and the 3D experiment, respectively.

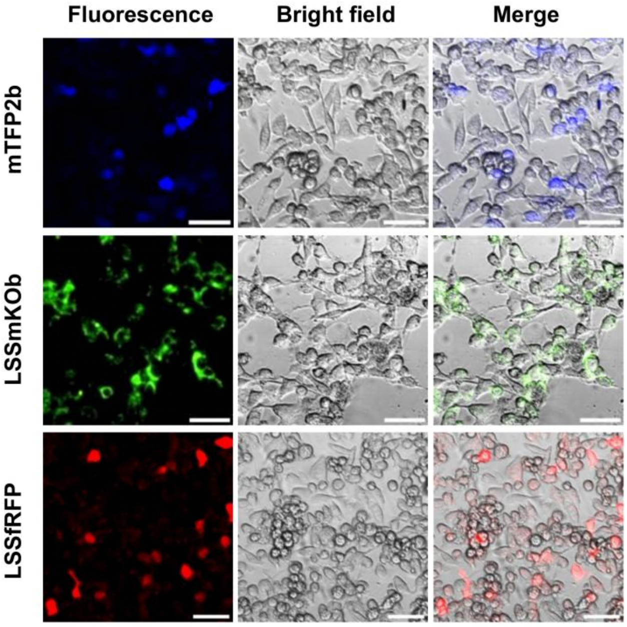

Supplementary Figure 10 Maturation of mTFP2b, LSSmKOb and LSSfRFP in B16 living cells. Fluorescence images were taken after 12 hours transfection. Scale bar, 50 μm.

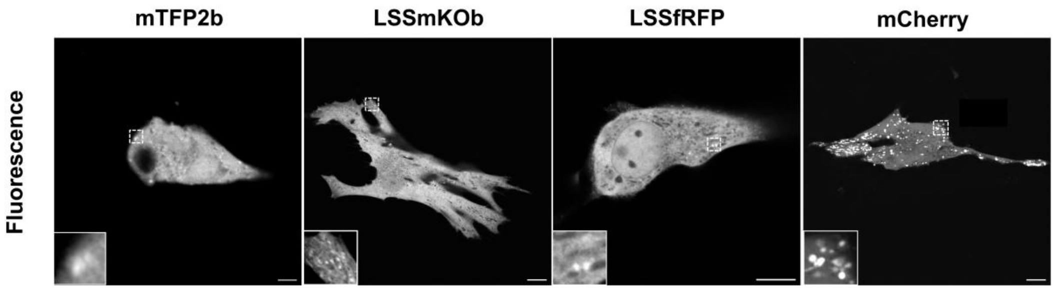

Supplementary Figure 11 Fluorescence images of B16 cells expressing mTFP2b, LSSmKOb, LSSfRFP and mCherry. B16 cells were transiently transfected with FPs and imaged 5 days after transfection. Imaging was performed with an inverted confocal microscope (FV3000, Olympus, Japan) equipped with an oil 100× NA1.45 objective, a 405 nm laser for mTFP2b, LSSmKOb and LSSfRFP, a 561 nm laser for mCherry, and four emission collection channels (470-510 nm for mTFP2b, 553-602 nm for LSSmKOb, 635-735 nm for LSSfRFP and 570-670 nm for mCherry). Typical fluorescent puncta are shown in the left bottom corner of each image. Scale bar, 5 µm.

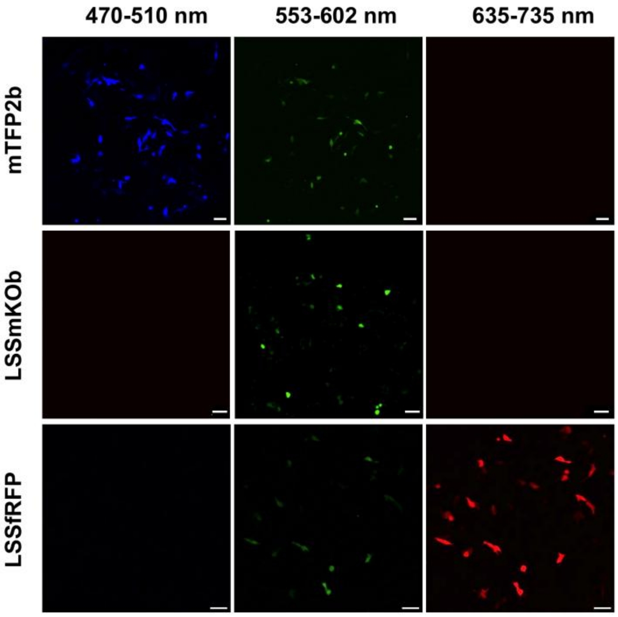

Supplementary Figure 12 Spectral cross-talk between FPs in UFObow. Representative fluorescence images from three emission channels for each FP in B16 living cells. All three images in each column have same contrast. Scale bar, 20 μm.

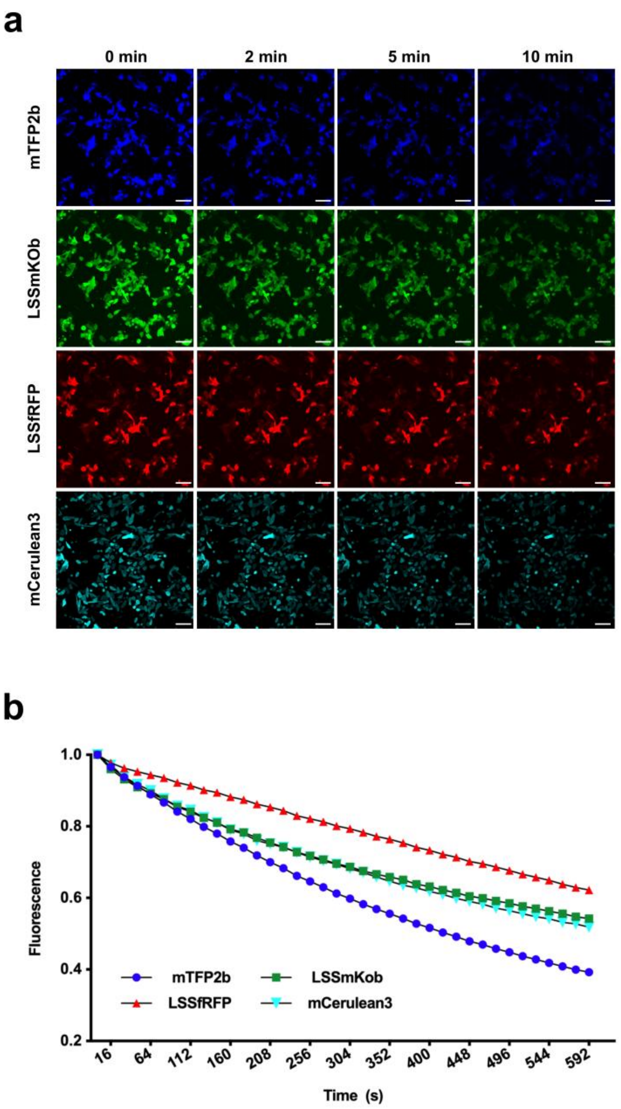

Supplementary Figure 13 Photostability of mTFP2b, LSSmKOb and LSSfRFP in B16 living cells. (a) Fluorescence images of FPs at different illumination time-points. **(b)** Fluorescence kinetics of FPs under continuous illumination. Scale bar, 50 μm.

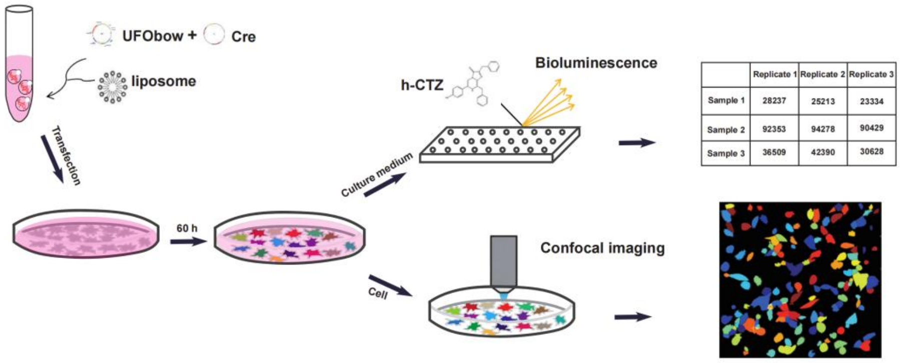

Supplementary Figure 14 Experimental workflow of characterization of UFObow in living cells. h-CTZ (h-coelenterazine) is the substrate of Nluc.

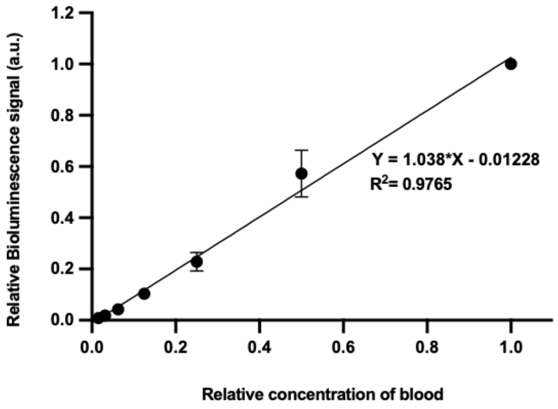

Supplementary Figure 15 The BL signal intensity is linearly proportional to the amount of secNluc. The blood from a UFObow mouse was gradient diluted into 2, 4, 8, 16, 32, 64, 128 and 256 folds, whose relative concentrations are 1/2, 1/4, 1/8, 1/16, 1/32, 1/64, 1/128 and 1/256, respectively. The bioluminescence signal at each certain concentration was normalized to that of undiluted blood.

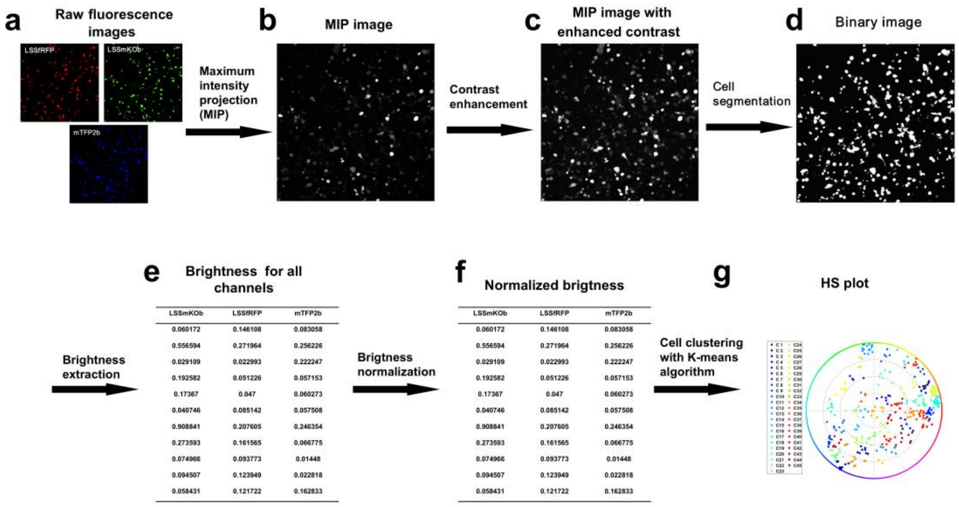

Supplementary Figure 16 Data processing pipeline for determining color complexity. (a) Raw fluorescence images from three fluorescence channels. (**b)** The images in (a) are integrated into one grayscale image using the maximum intensity projection method in Fiji. **(c)** The image in (b) is enhanced in contrast to highlight the difference between cells and background. (**d)** The Otsu cell segmentation algorithm is used to segment cells in (c) and generate binary images (fluorescence and background are set to 1 and 0, respectively). Notably, if the segmented image has cell adhesions, a watershed algorithm will be applied to separate the adhesions. If the segmented image has non-cell dots, a corrosion operation will be used to remove the dots. **(e)** Apply the binary image to the fluorescence images in (a) to obtain the fluorescence intensity in each channel and the number of fluorescent cells. (**f)** Map the fluorescence intensity of each channel to the [0,1]. **(g)** Apply the K-means algorithm to the data in (f) to obtain color clusters in the HS color model. The K-means clustering is performed by setting an initial number of clusters to 30 or 35 (approximately half of the theoretical color complexity in the case of 10 copies of the UFObow DNA in cells) and then running the K-means algorithm for 100 iterations.

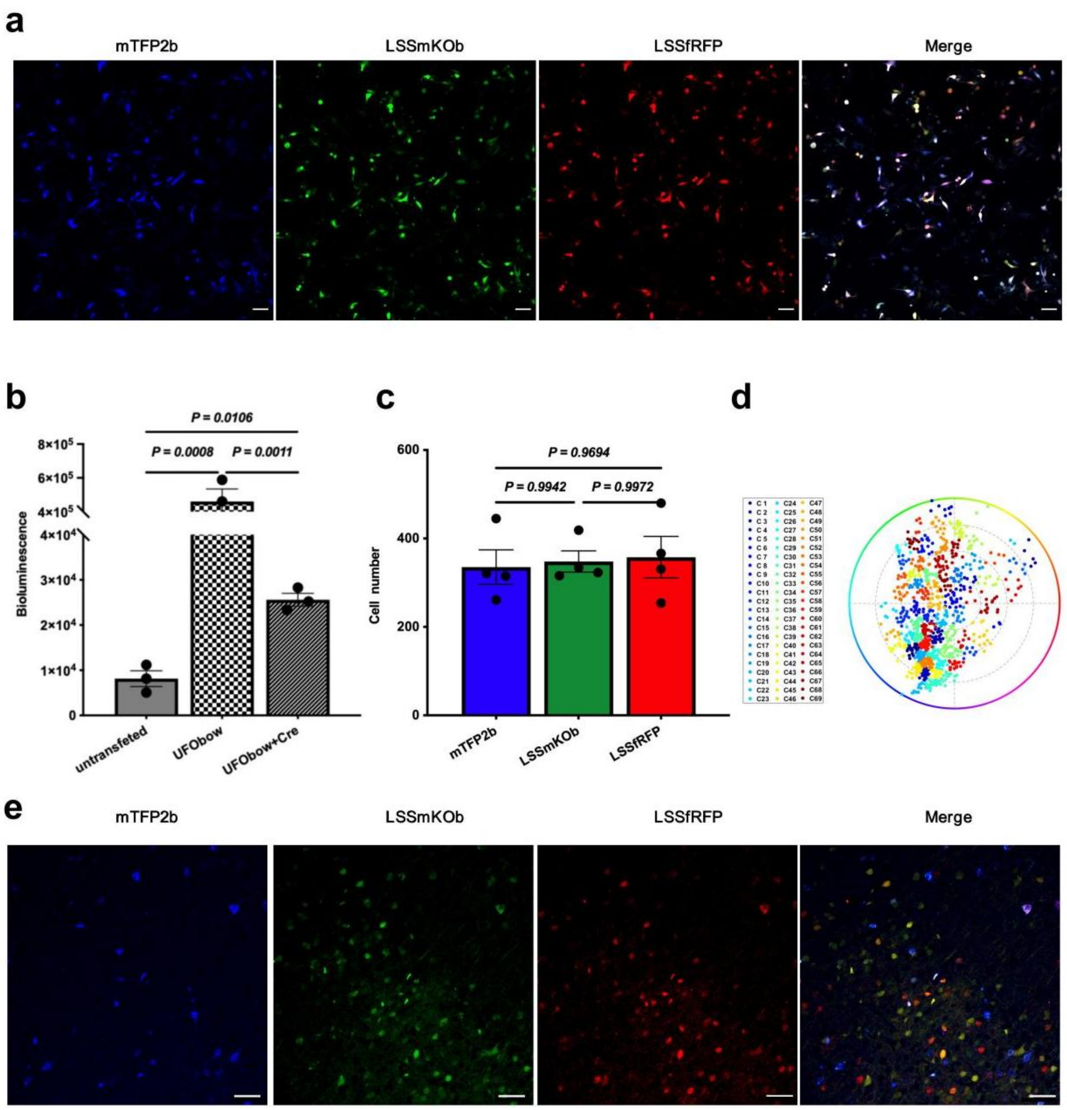

Supplementary Figure 17 Characterization of UFObow in living B16 cells. (a) Fluorescence images of B16 cells expressing UFObow and Cre. Scale bar, 100 μm. **(b)** Bioluminescence detection of Nluc in B16 cells expressing UFObow only or both UFObow and Cre. *P* values were calculated by one-way ANOVA with Tukey's multiple comparisons post-test. **(c)** Numbers of B16 cells expressing each FP: 336 ± 67 for mTFP2b, 348 ± 41 for LSSmKOb and 358 ± 81 for LSSfRFP. *P* values were calculated by one-way ANOVA with Tukey's multiple comparisons post-test. **(d)** The HS plot of cell clusters for cells in (b). Each dot in the plot represents a cell, and dots with same pseudo-color that is given by the clustering algorithm are within a cluster. 69 clusters (C1-C69) were achieved from 1041 cells. The SD and R values are 7.99% and 21.31%, respectively.

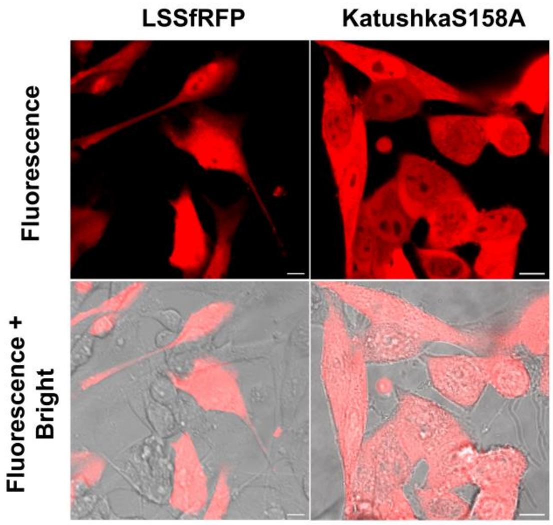

Supplementary Figure 18 Fluorescence images of B16 cells expressing LSSfRFP and Katushka-S158A. B16 cells were transiently transfected with FPs and imaged 5 days after transfection. Imaging was performed with an inverted confocal microscope (FV3000, Olympus, Japan) equipped with an oil 100× NA1.45 objective, a 405 nm laser for LSSfRFP, a 594 nm laser for Katushka-S158A, and two emission collection channels (635-735 nm for LSSfRFP and 600-690 nm for Katushka-S158A). Scale bar, 10 µm.

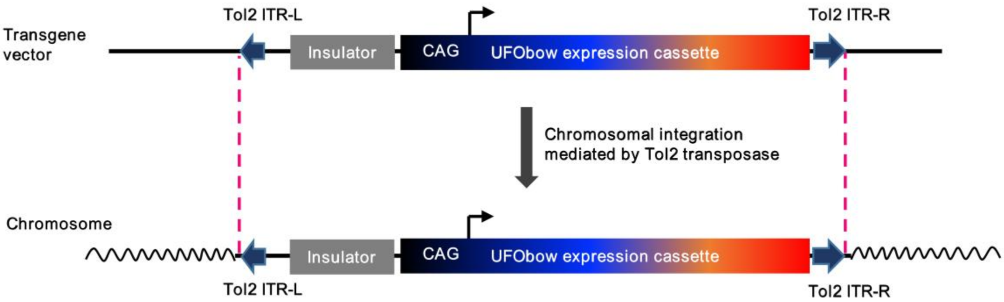

Supplementary Figure 19 Knock-in of UFObow using Tol2 transposase. The whole expression cassette of UFObow is subcloned into between two Tol2 inverted terminal repeats (ITR-L and ITR-R) in a transgene vector. An Insulator is placed right before the CAG promoter to protect UFObow from undesirable regulatory influences. In the presence of Tol2 transposase, the UFObow cassette is randomly integrated into the genome.

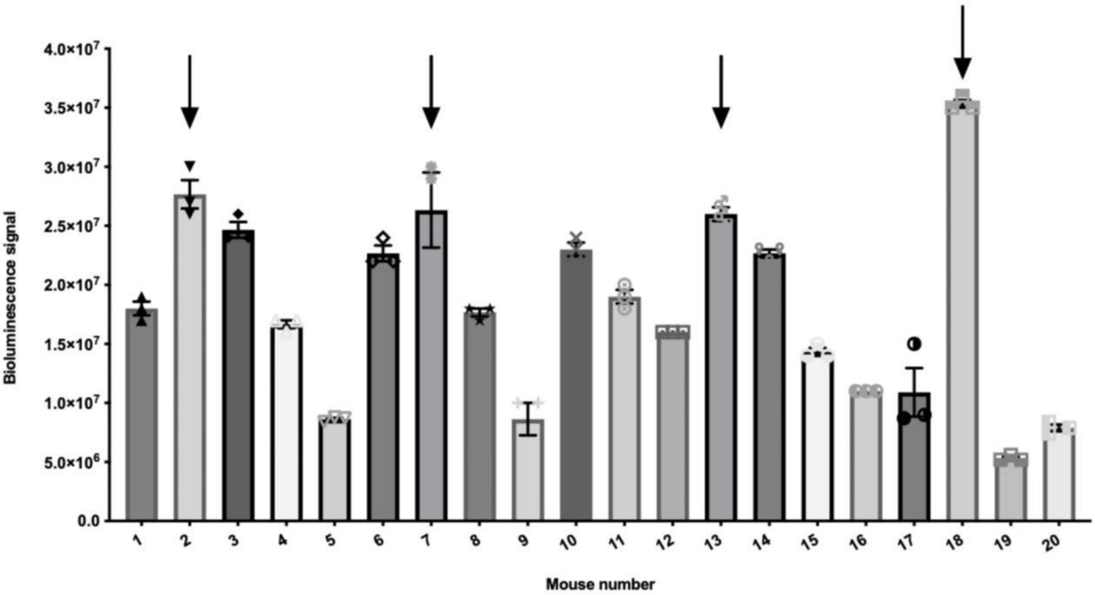

Supplementary Figure 20 Detection of Nluc in the blood of UFObow transgenic mice. The black and gray bars are males and females, respectively. Mice with high bioluminescence signal are indicated by black arrows and used in this study.

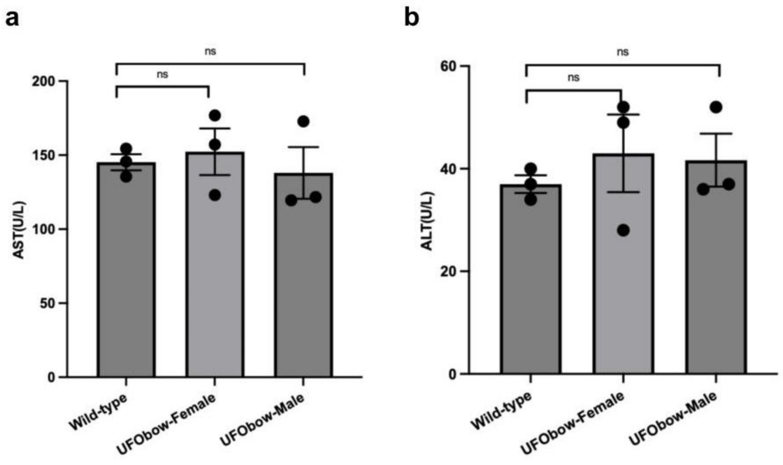

Supplementary Figure 21 Detection of aspartate aminotransferase (AST) and alanine aminotransferase (ALT) in UFObow mice. Enzyme activities were detected by automatic biochemical analyzer (3100E, Hitachi, Japan) with 15 μL of blood serum and reagent kits (H002 for AST and H001 for ALT, MedicalSystem, China). Data are presented as mean ± SEM (n=3). *P* values were calculated by one-way ANOVA with Tukey's multiple comparisons post-test. ns: not significant.

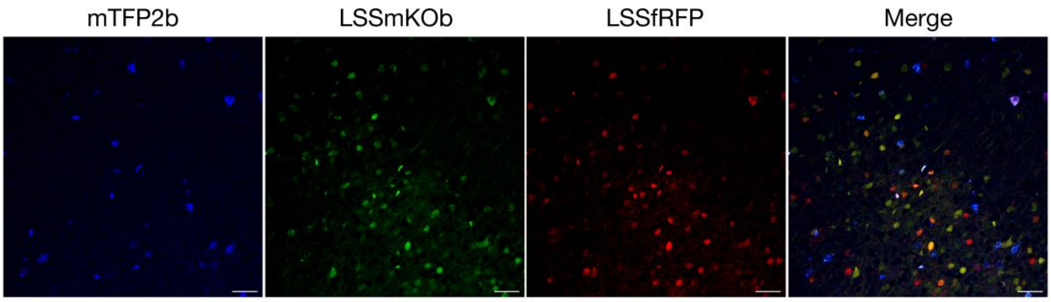

Supplementary Figure 22 Multicolor imaging of UFObow under two-photon excitation. Brain slices of the CX3CR1^{cre}:UFObow mouse were imaged using 860 nm excitation light and three emission collection channels: 470-510 nm for mTFP2b, 553-602 nm for LSSmKOb, and 635-735 nm for LSSfRFP. Imaging was performed on a two-photon microscope (Nikon, Ni-E, Japan) equipped with a water 25× NA1.1 objective (CFI Plan Apo lambda) and an ultrafast femtosecond pulsed laser (Mai Tai DeepSee, Spectra Physics) with a tunable 690-1040 nm wavelength range. Scale bar, 100 µm.

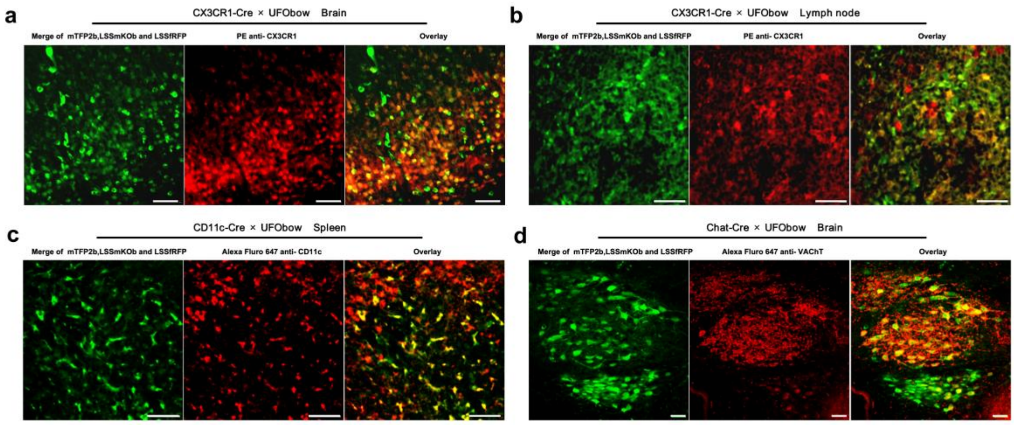

Supplementary Figure 23 Expression of UFObow in specific types of cells in different tissues of transgenic mice co-expressing UFObow and Cre. Each panel consists of three fluorescence images of a tissue slice: FP fluorescence (left), immunofluorescence (middle), and an overlay of FP fluorescence with immunofluorescence. FP fluorescence images are grayscale versions of 'RGB merge' images in Figure 3. FP fluorescence and immunofluorescence are shown in pseudocolor with green in red, respectively. **(a, b)** The representative fluorescence images of brain and lymph node slices of the CX3CR1^{cre}:UFObow mouse. The PE anti-CX3CR1 antibody labeled CX3CR1-positive cells. Scale bar in the (a), 100 µm. Scale bar in (b), 50 µm. **(c)** The representative fluorescence images of a spleen slice of the CD11c^{cre}:UFObow mouse. The Alexa Fluor 647 anti-CD11c antibody labeled CD11c-positive cells. Scale bar, 50 µm. **(d)** The representative fluorescence images of a brain slice of the Chat^{cre}:UFObow mouse. The Alexa Fluor 647 anti-VAChT antibody labeled VAChT-positive cells. Scale bar, 50 µm.

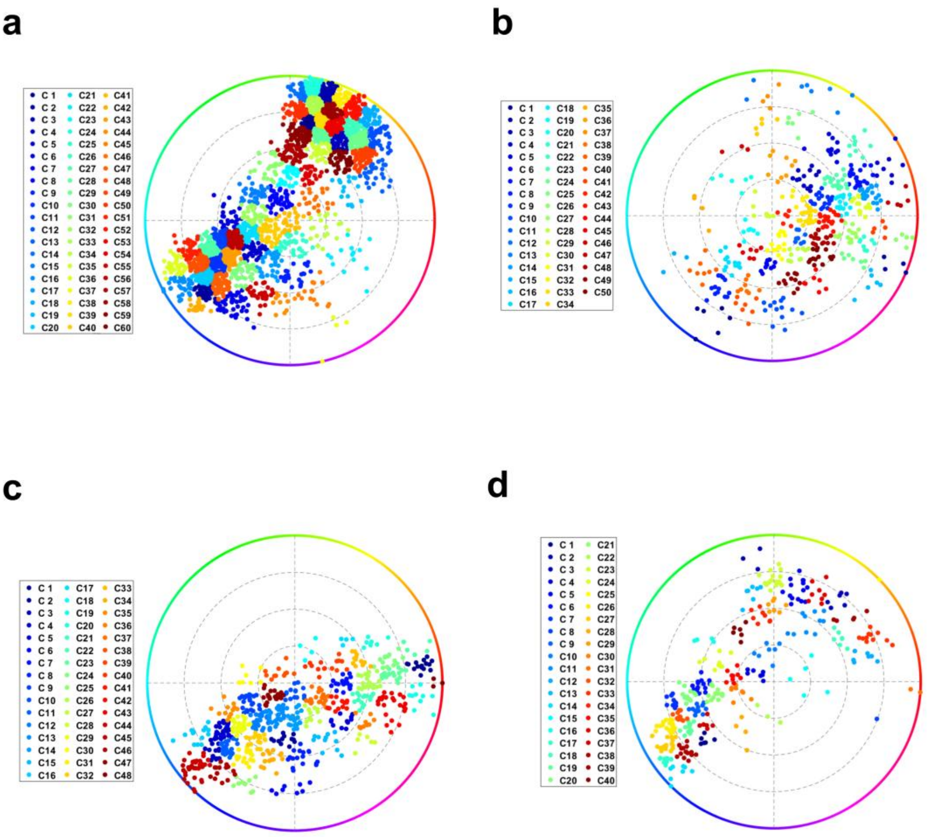

Supplementary Figure 24 HS plots of cell clusters for different tissues of transgenic mice co-expressing UFObow and Cre. (a, b) HS plots for the brain and lymph node of CX3CR1^{cre}:UFObow mouse with 60 (SD = 7.8%, R = 20.21%) and 50 clusters (SD = 7.98%, R = 22.86%) from 3006 and 483 cells, respectively. **(c)** The HS plot for spleen of CD11c^{cre}:UFObow mouse. 48 clusters were achieved from 713 cells (SD = 7.98%, R = 21.10%). **(d)** The HS plot for the cerebellum of Chat^{cre}:UFObow mouse. 40 clusters were achieved from 354 cells (SD = 7.55%, R = 22.77%).

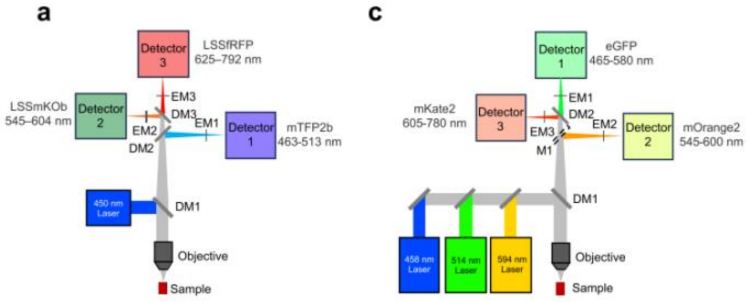

UFObow			
	Simultaneous Imaging		
FP	mTFP2b	LSSmKOb	LSSfRFP
Excitation	450 nm		
Channel	463-513 nm	545–604 nm	625–792 nm

Brainbow3			
	1st Imaging		2nd Imaging
FP	eGFP	mKate2	mOrange2
Excitation	458 nm	594 nm	514 nm
Channel	465-580 nm	605-780 nm	545-600 nm

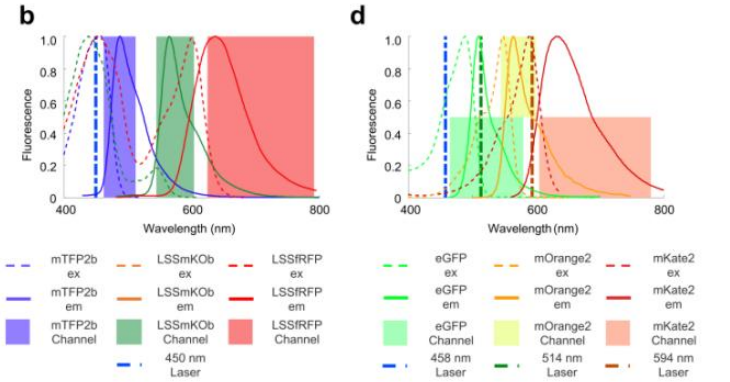

Supplementary Figure 25 Optical setups for imaging UFObow and Brainbow3. (a) Schematic diagram of optical setup of the modified cryo-fMOST system for imaging the UFObow-labeled samples. DM1: FF458-Di02-36×36 (Semrock, USA); DM2: FF520-Di02-25×36 (Semrock, USA); DM3: FF605-Di02-25x36 (Semrock, USA); EM1: FF01-488/50-25 (Semrock, USA); EM2: FF01-575/59-25 (Semrock, USA); EM3: FF01-709/167-25 (Semrock, USA). **(b)** Fluorescence spectra for FPs in UFObow with a 405 nm excitation laser and 3 emission channels. **(c)** Schematic diagram of the optical setup for FPs in Brainbow3. DM1 fixed dichroic mirror combinations of DM455+514/594. **(d)** Fluorescence spectra for FPs in Brainbow3 with 3 excitation lasers and 3 emission channels. DM, dichroic mirror; EM, emission filter; M, flip mirror.

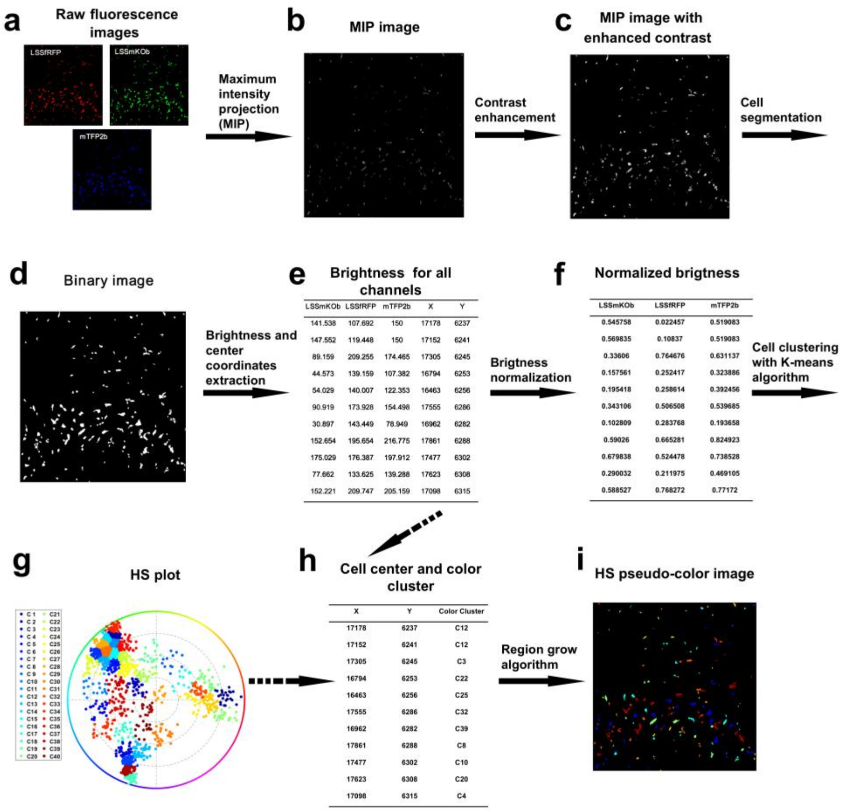

Supplementary Figure 26 Data processing pipeline for generating HS pseudo-color images. **(a-g)** The whole process for calculating the color complexity in the HS color model. The only difference between this process and Supplementary Figure 14 is that the spatial coordinate of each cell center (X, Y) is also extracted from (d) and displayed in (e). **(h)** Assign all colors in (g) to all cell centers (or seed points) in (e) with one color per seed point. **(i)** Generation of HS pseudo-color images. The region grow algorithm is performed on each seed point to get the corresponding cell colored with same color as the seed point.

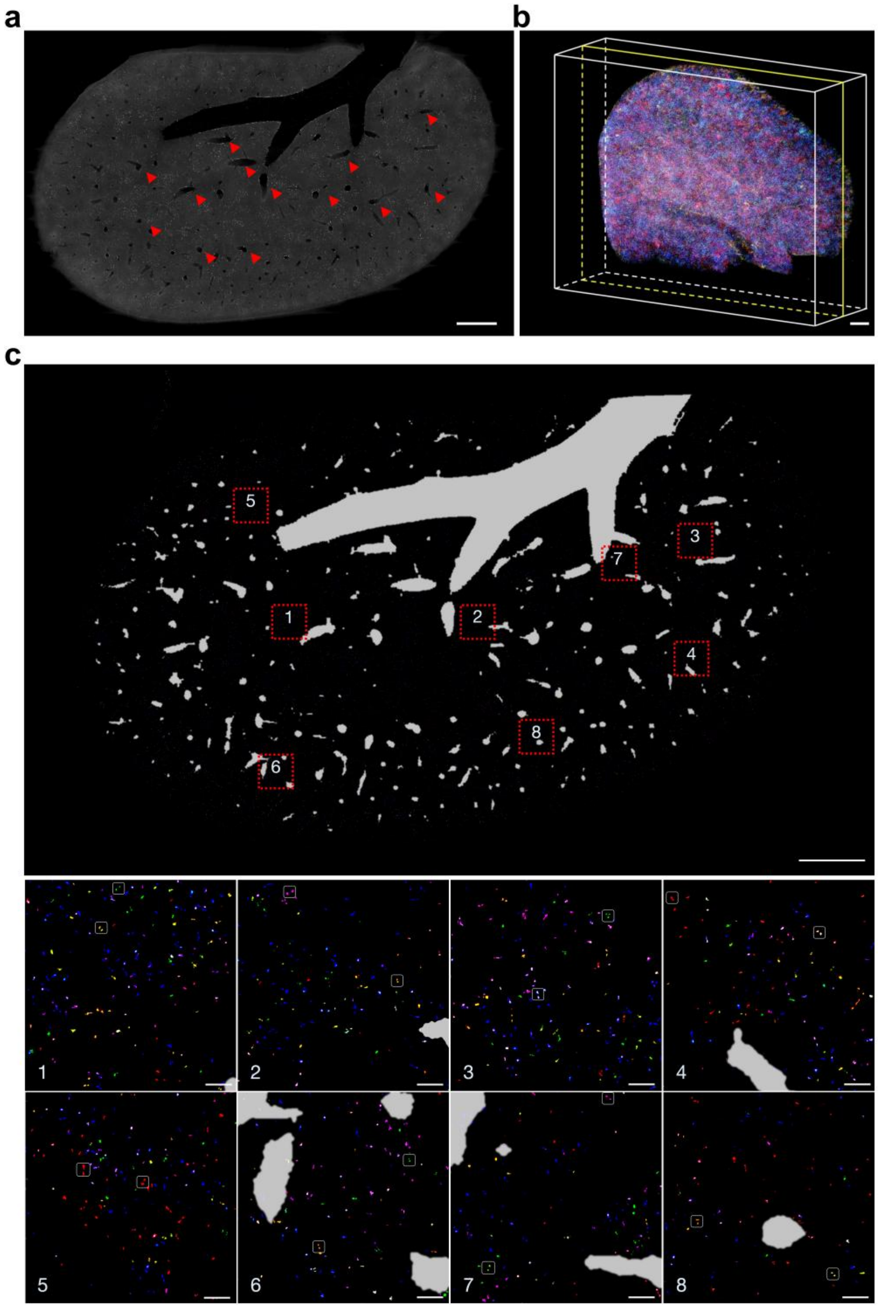

Supplementary Figure 27 MPs proliferate in the liver lobe. (a) Fluorescence image of the typical slice in the mTFP2b channel. Blood vessels, which appear as dark spots, are indicated by red arrows. The transverse stripes in the image were generated during line scan imaging due to luminance deviation, which were removed by brightness correction prior to 3D reconstruction. Scale bar, 500 μm. **(b)** A 3D view of the spatial distribution of MPs only in Figure 4a. Scale bar, 1 mm. The rectangles with yellow lines indicate the slice in (a). **(c)** Fluorescence merged images of combined six slices. Top and bottom panels are RGB merged-color images and HS pseudo-color images, respectively. The spectral cross-talk in (c) has been corrected. Two adjacent MP cells with the same color are framed by a square box. Scale bar in the large images, 1000 μm. Scale bar in the enlarged images, 100 μm. It is noted that all images in (c) are MIP images.

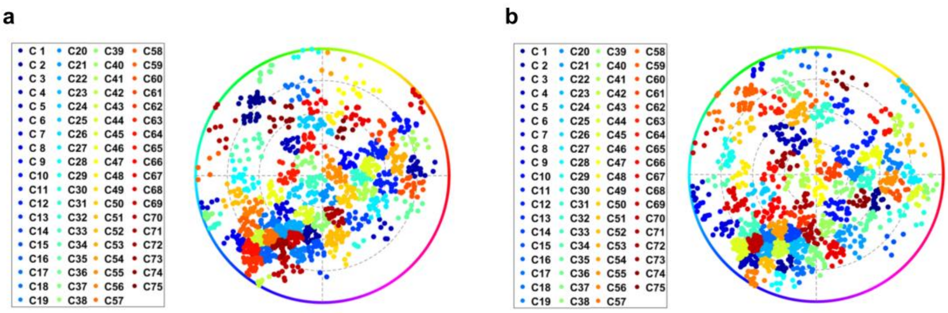

Supplementary Figure 28 Hue-Saturation (HS) plots of cell clusters in the CX3CR1^{cre}:UFObow liver. (a) and **(b)** are HS plots in the presence and absence of spectral cross-talk. Each dot in the plot represents a cell, and dots with same color that is given by the clustering K-means algorithm are within a cluster. 75 clusters (C1-C75) were achieved from 1332 cells. The angle between a given dot and the center of the circle represents the hue (0-360°) and the distance from the center of the circle to a given dot represents the saturation (0-100%). In the presence of spectral cross-talk (a), the intra-cluster dispersion (SD) and inter-cluster distance (R) are 7.67% and 20.08%, respectively. In the absence of spectral cross-talk (b), the SD and R are 7.72% and 20.51%, respectively.

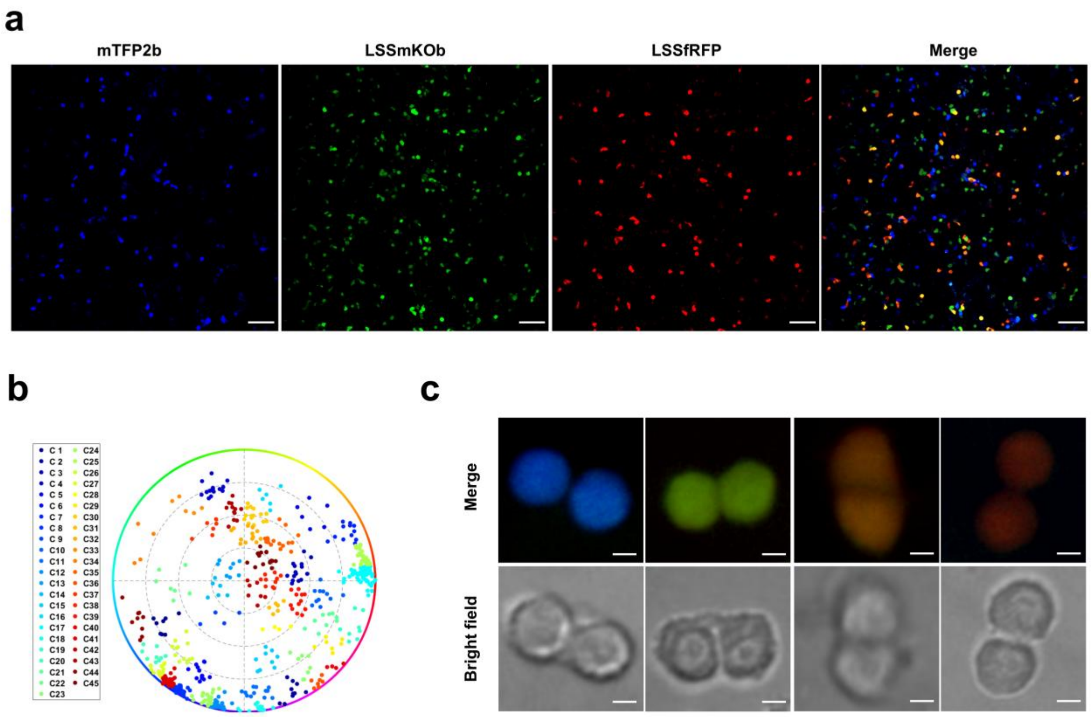

Supplementary Figure 29 Characterization of UFObow-B16 cells. (a) Fluorescence images of stable UFObow-B16 cells. Scale bar in large images, 200 μm. Scale bar in the enlarged image, 10 μm. **(b)** The HS plot of cell clusters for cells in (a). 45 clusters were achieved from 622 cells (SD = 7.6%， R = 21.2%). **(c)** Fluorescence imaging of dividing UFObow-B16 cells. Scale bar, 10 μm.

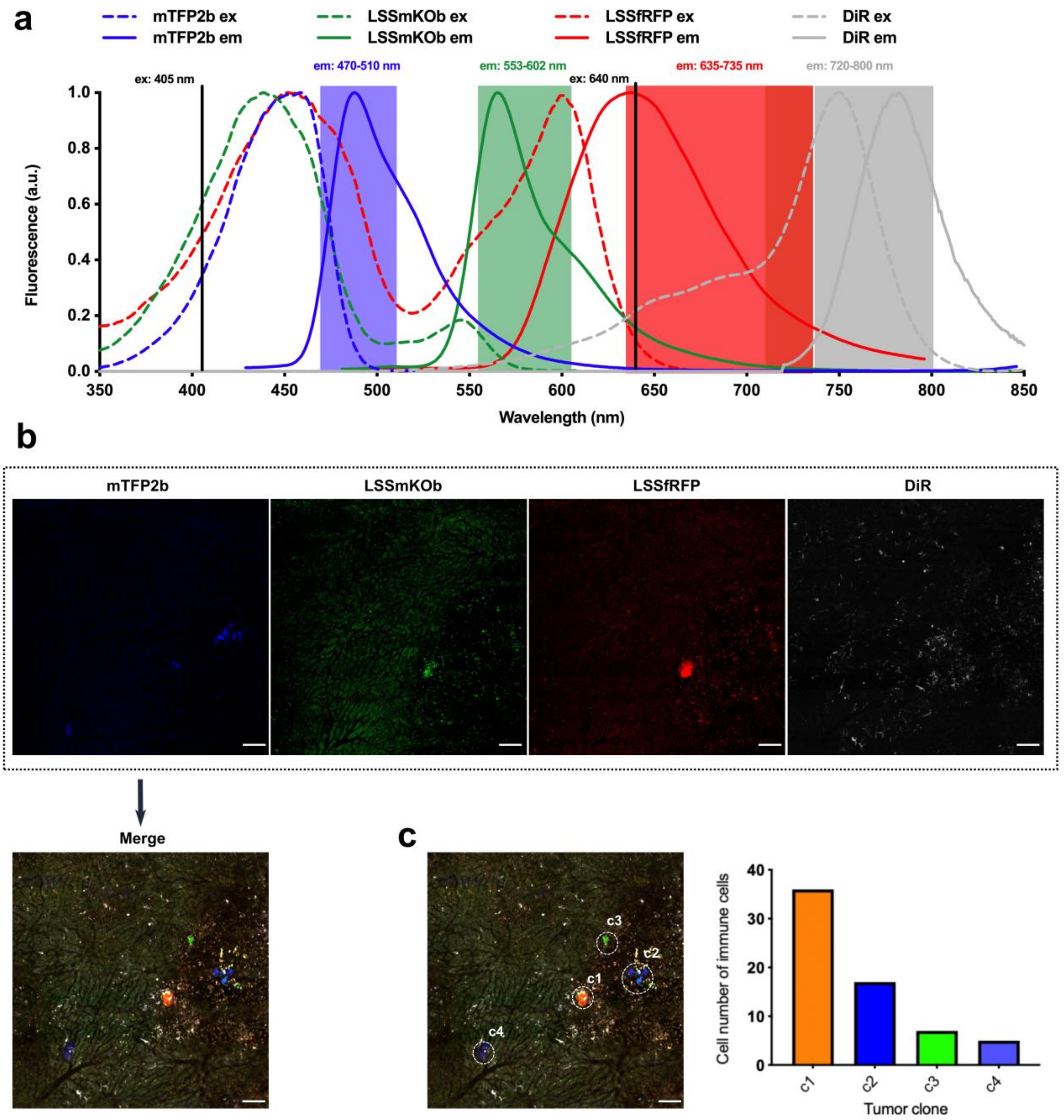

Supplementary Figure 30 Multicolor imaging of tumor clones and immune cells. (**a**) Fluorescence spectra of FPs in UFObow and DiR with fluorescence channels. The fluorescence of DiR is recorded from 720 to 800 nm under 640 nm excitation. (**b**) Fluorescence imaging of UFObow-B16 cells and nanopomegranate-labeled monocyte-macrophages in the intact liver via DAW. After 7 days intrasplenic injection of UFObow-B16 cells, nanopomegranate was intravenously injected into a mouse to label KCs and monocyte-macrophages in the liver 2 hours before intravital imaging. The fluorescence intensity of LSSfRFP in the DiR channel accounts for 7.0% of that of DiR in the DiR channel. Scale bar, 100 μm. (**c**) Cell number of immune cells (KCs and monocytes/macrophages) recruited by four tumor clones (c1-c4) in (b).

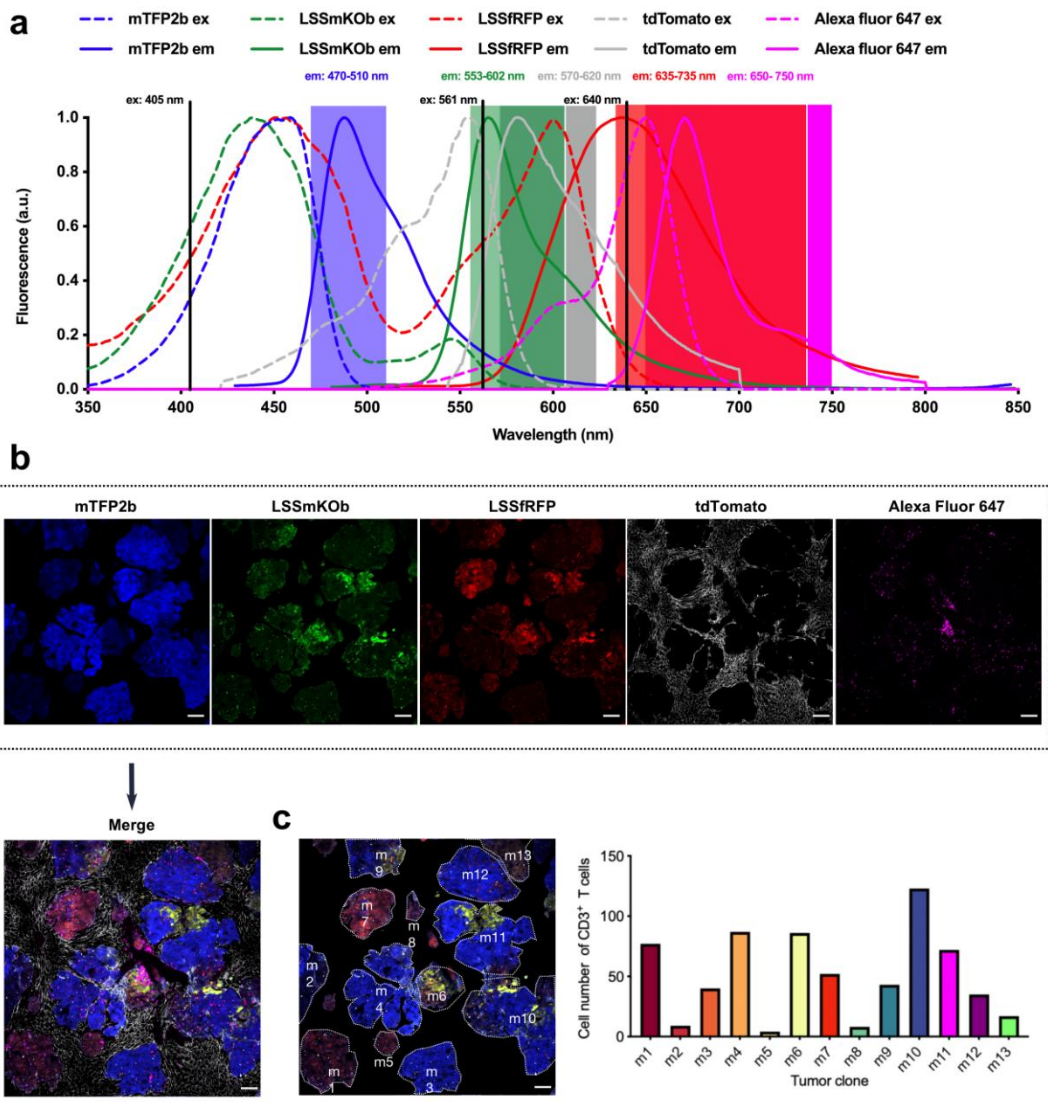

Supplementary Figure 31 Multicolor imaging of tumor clones, blood vessels, and CD3$^+$ T cells. **(a)** Fluorescence spectra of UFObow, tdTomato and Alexa Fluor 647 with fluorescence channels. The fluorescence of tdTomato and Alexa Fluor 647 are recorded from 570 to 620 nm under 561 nm excitation and 650-750 nm under 640 nm excitation, respectively. **(b)** Fluorescence imaging of UFObow-B16 cells, tdTomato-labeled vascular endothelial cells, and Alexa Fluor 647-labeled CD3$^+$ T cells in liver slice. After 11 days intrasplenic injection of UFObow-B16 in mT/mG transgenic mouse, the liver was sliced and then stained with Alexa Fluor 647 anti-CD3 antibody. The fluorescence intensity of LSSfRFP in the tdTomato channel accounts for 1.1% of that of tdTomato in the tdTomato channel. The fluorescence intensity of LSSfRFP in the Alex Fluor 647 channel accounts for 4.1% of that of Alex Fluor 647 channel in the Alex Fluor 647 channel. The fluorescence intensity of LSSmKOb in the tdTomato channel accounts for 1.2% of that of tdTomato in the tdTomato channel. Scale bar, 50 μm. **(c)** Cell number of CD3+ cells recruited by thirteen tumor clones (m1-m13).

Supplementary Movie 1: A 3D view of MPs (merged RGB color) and blood vessels (white) in an intact liver lobe. Part I: Animation of the 3D distribution of MPs (1762 RGB merged-color images) of the liver lobe. Part II: Animation of the 3D structure of vessels (1762 images) of the liver lobe. Part III: Display of maximum-intensity-projection (MIP) images. Each MIP image contains 8 consecutive RGB merged-color images.

Table S1. Photophysical properties of FPs.

FP	EC[a]	QY[b]	Brightness[c]	Ex peak (nm)	Em peak (nm)
mTFP	64	0.85	54.4	462	492
mTFP2b	54	0.74	40.0	459	488
LSSmOrange	52	0.45	23.4	437	572
LSSmKOb	44	0.49	21.6	439[d]	566
LSSfRFP	46	0.18	8.3	452[e]	637

[a]extinction coefficient ($mM^{-1}cm^{-1}$)

[b]quantum yield

[c]calculated as the product of EC and QY

[d]the secondary excitation peak is at 545 nm, which indicates the deprotonated form of LSSmOrange

[e]the secondary excitation peak is at 603 nm, which is from dCardinal.

Table S2. A list of PCR primers for constructing pUFObow and PB-UFObow

Primer	Sequence (5'-3')
	pUFObow
pUFObow-P1	TTTTGGCAAAGAATTCTCCTAAGGTAGCGAACCTAGG
pUFObow-P2	CGCTTGTGGAGAAGGAGTTCATGGTGGCGATATCAGATAACTTCG
secNluc-F	ATGAACTCCTTCTCCACAAGCG
secNluc-R	CGCCAGAATGCGTTCGCAC
pUFObow-P5	GTGCGAACGCATTCTGGCGTGAAGCTTAATTAGCTGAGCTTGGA
pUFObow-P6	CATGGTGGCGGATCCAGATAACTTCGTATAATGTATGCTATACG
LSSmKOb-F	GTTATCTGGATCCGCCACCATGGTGAGTAAGGGAGAGGCT
LSSmKOb-R	CAGAGGTTGATTACTGCAGTCACTTGTACAGCTCGTCCATGCC
pUFObow-P9	GTGACTGCAGTAATCAACCTCTGGATTACAAAATTTGTG
pUFObow-P10	GGTGGCTCCGGACGATAACTTCGTATAAAGTATCCTATAC
mTFP2b-F	AGTTATCGTCCGGAGCCACCATGGTTAGCAAGGGCGAGGAG
mTFP2b-R	GAGGTTGATTAGAGCTCATTTGTACAGCTCATCCATGCTGTCGGT
pUFObow-P13	CTGTACAAATGAGCTCTAATCAACCTCTGGATTACAAAATTTGTG

pUFObow-P14	ACCATGGTGGCGGTACCTGATAACTTCGTATAAGGTATACTATACG
LSSfRFP-F-1	CAGGTACCGCCACCATGGTGTCTAAGGGCGAGGAG
LSSfRFP-R-1	GTGCCTCCCACATTTGTGTGC
LSSfRFP-F-2	GCACACAAATGTGGGAGGCAC
LSSfRFP-R-2	CCAGAGGTTGATTATGCATCACTTGTACAGCTCATCCATGCC
pUFObow-P19	CAAGTGATGCATAATCAACCTCTGGATTACAAAATTTGTG
pUFObow-P20	CCTTTTGCTCACATGTAGGAAGGACCTCGAGCTAGTAC
pcDNA3.1-mTFP2b, pcDNA3.1-LSSmKOb and pcDNA3.1-LSSfRFP	
mTFP2b-F	CGCGGATCCGCCACCATGGTTAGCAAGGGCGAG
mTFP2b-R	CCGGAATTCTCATTTGTACAGCTCATCCATGCT
LSSmKOb-F	GGATCCATGGTGAGTAAGGGAGAGGCTGTC
LSSmKOb-R	GAATTCCTTGTACAGCTCGTCCATGCCGAG
LSSfRFP-F	GGATCCATGGTGTCTAAGGGCGAGGAGCTG
LSSfRFP-R	GAATTCCTTGTACAGCTCATCCATGCCATTCAGCTTGTGGCC
PB-UFObow	
PB-F	CTTAAGCCTAGGCATGCCGGAGGTGGCGGTAAAGGGT
PB-R	GAATTCTAACTATAACGGTCCTAAGGTAGCGATTACTTGTACAGCTCGTCCATGCCG

References

1. Chu J*, et al.* A bright cyan-excitable orange fluorescent protein facilitates dual-emission microscopy and enhances bioluminescence imaging in vivo. *Nat Biotechnol* **34**, 760-767 (2016).
2. Qi S*, et al.* Long-term intravital imaging of the multicolor-coded tumor microenvironment during combination immunotherapy. *Elife* **5**, 14756 (2016).
3. Yu X*, et al.* Immune modulation of liver sinusoidal endothelial cells by melittin nanoparticles suppresses liver metastasis. *Nat Commun* **10**, 574 (2019).
4. Deng D*, et al.* A drawer-type abdominal window with an acrylic/resin coverslip enables long-term intravital fluorescence/photoacoustic imaging of the liver. *Nanophotonics* **10**, 3369-3381 (2021).
5. Lin Q*, et al.* Self-Assembled "Off/On" nanopomegranate for in vivo photoacoustic and fluorescence imaging: strategic arrangement of Kupffer cells in mouse hepatic lobules. *ACS Nano* **13**, 1526-1537 (2019).
6. Dai B*, et al.* Intravital molecular imaging reveals that ROS-caspase-3-GSDME-induced cell punching enhances humoral immunotherapy targeting intracellular tumor antigens. *Theranostics* **12**, 7603-7623 (2022).
7. Deng L*, et al.* Cryo-fluorescence micro-optical sectioning tomography for volumetric imaging of various whole organs with subcellular resolution. *iScience* **25**, 104805 (2022).
8. Xu D, Tian Y. A comprehensive survey of clustering algorithms. *Annals of Data Science* **2**, 165-193 (2015).
