# Supplementary material for: UFObow: A single-wavelength excitable Brainbow for simultaneous multicolor ex-vivo and in-vivo imaging of mammalian cells
Source: Commun Biol. 2024 Apr 1;7:394. doi: 10.1038/s42003-024-06062-3 (PMC10984974; doi:10.1038/s42003-024-06062-3)
Supplement: Supplementary file 2 — Description of Additional Supplementary Files [file 42003_2024_6062_MOESM2_ESM.pdf]

Description of Additional Supplementary Files

File name: Supplementary Data 1
Description: The source data behind the graphs in the paper.

File name: Supplementary Movie 1
Description: A 3D view of MPs and blood vessels in an intact liver lobe.
