# Supplementary material for: UFObow: A single-wavelength excitable Brainbow for simultaneous multicolor ex-vivo and in-vivo imaging of mammalian cells
Source: Commun Biol. 2024 Apr 1;7:394. doi: 10.1038/s42003-024-06062-3 (PMC10984974; doi:10.1038/s42003-024-06062-3)
Supplement: Supplementary file 5 — Reporting Summary [file 42003_2024_6062_MOESM5_ESM.pdf]

nature portfolio

Corresponding author(s): Jun Chu and COMMSBIO-23-1950B

Last updated by author(s): Jan 31, 2024

Reporting Summary

Nature Portfolio wishes to improve the reproducibility of the work that we publish. This form provides structure for consistency and transparency in reporting. For further information on Nature Portfolio policies, see our Editorial Policies and the Editorial Policy Checklist.

Statistics

For all statistical analyses, confirm that the following items are present in the figure legend, table legend, main text, or Methods section.

n/a	Confirmed	
☐	☒	The exact sample size (*n*) for each experimental group/condition, given as a discrete number and unit of measurement
☐	☒	A statement on whether measurements were taken from distinct samples or whether the same sample was measured repeatedly
☒	☐	The statistical test(s) used AND whether they are one- or two-sided<br>*Only common tests should be described solely by name; describe more complex techniques in the Methods section.*
☒	☐	A description of all covariates tested
☐	☒	A description of any assumptions or corrections, such as tests of normality and adjustment for multiple comparisons
☐	☒	A full description of the statistical parameters including central tendency (e.g. means) or other basic estimates (e.g. regression coefficient) AND variation (e.g. standard deviation) or associated estimates of uncertainty (e.g. confidence intervals)
☐	☒	For null hypothesis testing, the test statistic (e.g. *F*, *t*, *r*) with confidence intervals, effect sizes, degrees of freedom and *P* value noted<br>*Give P values as exact values whenever suitable.*
☒	☐	For Bayesian analysis, information on the choice of priors and Markov chain Monte Carlo settings
☒	☐	For hierarchical and complex designs, identification of the appropriate level for tests and full reporting of outcomes
☒	☐	Estimates of effect sizes (e.g. Cohen's *d*, Pearson's *r*), indicating how they were calculated

Our web collection on statistics for biologists contains articles on many of the points above.

Software and code

Policy information about availability of computer code

Data collection	The Olympus FV3000 confocal imaging system was used to collect fluorescence images of cells and tissue sections. The Molecular Devices FlexStation 3 multifunctional enzyme label instrument system was used to detect the bioluminescence signal. The fluorescence spectra were taken with a Tecan Infinite Pro 2000. The 3D imaging data was collected by a custom software.
Data analysis	The image processing was mainly completed in Fiji version 1.52, MATLAB version 2021a, and Imaris version 7.45.2. All statistical analyses were performed using GraphPad Prism version 7.2 (trial version). All FP structure figures were generated using PyMol (version 2.5.4, Schrödinger, LLC, Portland, USA) without raying.

For manuscripts utilizing custom algorithms or software that are central to the research but not yet described in published literature, software must be made available to editors and reviewers. We strongly encourage code deposition in a community repository (e.g. GitHub). See the Nature Portfolio guidelines for submitting code & software for further information.

Data

Policy information about availability of data

All manuscripts must include a data availability statement. This statement should provide the following information, where applicable:

- Accession codes, unique identifiers, or web links for publicly available datasets
- A description of any restrictions on data availability
- For clinical datasets or third party data, please ensure that the statement adheres to our policy

The authors declare that the main data supporting the findings of this study are available within the article and its Supplementary Materials. Extra data are available from the corresponding author upon reasonable request.

Research involving human participants, their data, or biological material

Policy information about studies with human participants or human data. See also policy information about sex, gender (identity/presentation), and sexual orientation and race, ethnicity and racism.

Reporting on sex and gender	*Use the terms sex (biological attribute) and gender (shaped by social and cultural circumstances) carefully in order to avoid confusing both terms. Indicate if findings apply to only one sex or gender; describe whether sex and gender were considered in study design; whether sex and/or gender was determined based on self-reporting or assigned and methods used. Provide in the source data disaggregated sex and gender data, where this information has been collected, and if consent has been obtained for sharing of individual-level data; provide overall numbers in this Reporting Summary. Please state if this information has not been collected. Report sex- and gender-based analyses where performed, justify reasons for lack of sex- and gender-based analysis.*
Reporting on race, ethnicity, or other socially relevant groupings	*Please specify the socially constructed or socially relevant categorization variable(s) used in your manuscript and explain why they were used. Please note that such variables should not be used as proxies for other socially constructed/relevant variables (for example, race or ethnicity should not be used as a proxy for socioeconomic status). Provide clear definitions of the relevant terms used, how they were provided (by the participants/respondents, the researchers, or third parties), and the method(s) used to classify people into the different categories (e.g. self-report, census or administrative data, social media data, etc.) Please provide details about how you controlled for confounding variables in your analyses.*
Population characteristics	*Describe the covariate-relevant population characteristics of the human research participants (e.g. age, genotypic information, past and current diagnosis and treatment categories). If you filled out the behavioural & social sciences study design questions and have nothing to add here, write "See above."*
Recruitment	*Describe how participants were recruited. Outline any potential self-selection bias or other biases that may be present and how these are likely to impact results.*
Ethics oversight	*Identify the organization(s) that approved the study protocol.*

Note that full information on the approval of the study protocol must also be provided in the manuscript.

Field-specific reporting

Please select the one below that is the best fit for your research. If you are not sure, read the appropriate sections before making your selection.

☒ Life sciences ☐ Behavioural & social sciences ☐ Ecological, evolutionary & environmental sciences

For a reference copy of the document with all sections, see nature.com/documents/nr-reporting-summary-flat.pdf

Life sciences study design

All studies must disclose on these points even when the disclosure is negative.

Sample size	No sample size calculation was performed. Sample size was determined to be adequate based on the magnitude and consistency of measurable differences between groups.
Data exclusions	No data were excluded from the analyses in this study.
Replication	Every experiment was repeated once or more to ensure every major finding is highly repeatable.
Randomization	To prevent selection bias, the imaging area was randomly selected and imaged multiple times.
Blinding	Blinding was not involved in this study.

Reporting for specific materials, systems and methods

We require information from authors about some types of materials, experimental systems and methods used in many studies. Here, indicate whether each material, system or method listed is relevant to your study. If you are not sure if a list item applies to your research, read the appropriate section before selecting a response.

Materials & experimental systems

n/a	Involved in the study	
☐	☒	Antibodies
☐	☒	Eukaryotic cell lines
☒	☐	Palaeontology and archaeology
☐	☒	Animals and other organisms
☒	☐	Clinical data
☒	☐	Dual use research of concern
☒	☐	Plants

Methods

n/a	Involved in the study	
☒	☐	ChIP-seq
☒	☐	Flow cytometry
☒	☐	MRI-based neuroimaging

Antibodies

Antibodies used	PE anti-CX3CR1 (Clone SA011F11 Cat# 149005, BioLegend), Alexa Fluor 647 anti-CD11c (Clone N418 Cat# 117312, BioLegend), Alexa Fluor 647 anti-CD3 (Clone 17A2 Cat# 100209, BioLegend), goat anti-mouse VAChT (Clone ABN100, Sigma), and donkey anti-goat IgG(H+L) (Clone A21447, Sigma)
Validation	PE anti-CX3CR1 (Clone SA011F11 Cat# 149005, BioLegend), Application: FC (Flow Cytometry). The validation has been performed by the manufacturer: https://www.biolegend.com/en-us/products/pe-anti-mouse-cx3cr1-antibody-10376.<br>Alexa Fluor 647 anti-CD11c (Clone N418 Cat# 117312, BioLegend). Application: FC (Flow Cytometry). The validation has been performed by the manufacturer: https://www.biolegend.com/en-us/products/alexa-fluor-647-anti-mouse-cd11c-antibody-2703.<br>Alexa Fluor 647 anti-CD3 (Clone 17A2 Cat# 100209, BioLegend). Application: FC (Flow Cytometry). The validation has been performed by the manufacturer: https://www.biolegend.com/en-us/products/alexa-fluor-647-anti-mouse-cd3-antibody-2693.<br>Goat anti-mouse VAChT (Clone ABN100, Sigma). Application: ELISA and immunohistochemistry. The validation has been performed by the manufacturer:https://www.sigmaaldrich.cn/CN/zh/product/mm/abn100.<br>Donkey anti-goat IgG(H+L) (Clone A21447, Sigma). Application: Immunohistochemistry. The validation has been performed by the manufacturer: https://www.thermofisher.cn/cn/zh/antibody/product/Donkey-anti-Goat-IgG-H-L-Cross-Adsorbed-Secondary-Antibody-Polyclonal/A-21447.

Eukaryotic cell lines

Policy information about cell lines and Sex and Gender in Research

Cell line source(s)	HeLa and B16F10 (B16) melanoma cell lines were obtained from ATCC and the BOSTER company (Wuhan, China), respectively.
Authentication	These cell lines were authenticated using short tandem repeat (STR) profiling.
Mycoplasma contamination	These cell lines were mycoplasma-negative as determined by screening using the MycoProbe Mycoplasma Detection Kit (R and D Systems, Minneapolis, MN).
Commonly misidentified lines<br>(See ICLAC register)	There are no commonly misidentified lines in this study.

Animals and other research organisms

Policy information about studies involving animals; ARRIVE guidelines recommended for reporting animal research, and Sex and Gender in Research

Laboratory animals	Wild type C57BL/6 mice<br>B6J.B6N(Cg)-Cx3cr1tm1.1(cre)Jung/J (CX3CR1-Cre, JAX: 025524)<br>B6.Cg-Tg(Itgax-cre)1-1Reiz/J (CD11c-Cre, JAX: 008068)<br>B6;129S6-Chattm2(cre)Lowl/J (Chat-Cre, JAX: 006410)<br>B6.129(Cg)-Gt(ROSA)26ortm4(ACTB-tdTomato,-EGFP)Luo/J (mT/mG, JAX: 007676)<br>All mice were bred at the animal facility of WNLO-HUST under specific-pathogen-free (SPF) conditions and used at 6-10 weeks old. All animal experiments were approved by the Animal Experimentation Ethics Committee of Huazhong University of Science and Technology (IACUC Number: 843).
Wild animals	This study did not involve wild animals.
Reporting on sex	The findings of this study can be applied without gender differences.

Field-collected samples	The study did not involve samples collected from the field.
Ethics oversight	All mice were bred at the animal facility of WNLO-HUST under specific-pathogen-free (SPF) conditions and used at 6-10 weeks old. All animal experiments were approved by the Animal Experimentation Ethics Committee of Huazhong University of Science and Technology (IACUC Number: 843).

Note that full information on the approval of the study protocol must also be provided in the manuscript.
